# Contrasting invertebrate immune defense behaviors caused by a single gene, the *Caenorhabditis elegans* neuropeptide receptor gene *npr-1*

**DOI:** 10.1186/s12864-016-2603-8

**Published:** 2016-04-11

**Authors:** Rania Nakad, L. Basten Snoek, Wentao Yang, Sunna Ellendt, Franziska Schneider, Timm G. Mohr, Lone Rösingh, Anna C. Masche, Philip C. Rosenstiel, Katja Dierking, Jan E. Kammenga, Hinrich Schulenburg

**Affiliations:** Department of Evolutionary Ecology and Genetics, Zoological Institute, University of Kiel, 24098 Kiel, Germany; Cologne Excellence Cluster for Cellular Stress Responses in Ageing-Associated Diseases (CECAD) and Systems Biology of Ageing, University of Cologne, Joseph-Stelzmann-Str. 26, 50931 Cologne, Germany; Laboratory of Nematology, Wageningen University, Wageningen, 6708 PB The Netherlands; Institute for Clinical Molecular Biology, University of Kiel, 24098 Kiel, Germany

**Keywords:** *Caenorhabditis elegans*, Pathogen avoidance behavior, Innate immunity, Immune specificity, QTL analysis

## Abstract

**Background:**

The invertebrate immune system comprises physiological mechanisms, physical barriers and also behavioral responses. It is generally related to the vertebrate innate immune system and widely believed to provide nonspecific defense against pathogens, whereby the response to different pathogen types is usually mediated by distinct signalling cascades. Recent work suggests that invertebrate immune defense can be more specific at least at the phenotypic level. The underlying genetic mechanisms are as yet poorly understood.

**Results:**

We demonstrate in the model invertebrate *Caenorhabditis elegans* that a single gene, a homolog of the mammalian neuropeptide Y receptor gene, *npr-1*, mediates contrasting defense phenotypes towards two distinct pathogens, the Gram-positive *Bacillus thuringiensis* and the Gram-negative *Pseudomonas aeruginosa*. Our findings are based on combining quantitative trait loci (QTLs) analysis with functional genetic analysis and RNAseq-based transcriptomics. The QTL analysis focused on behavioral immune defense against *B. thuringiensis*, using recombinant inbred lines (RILs) and introgression lines (ILs). It revealed several defense QTLs, including one on chromosome X comprising the *npr-1* gene. The wildtype N2 allele for the latter QTL was associated with reduced defense against *B. thuringiensis* and thus produced an opposite phenotype to that previously reported for the N2 *npr-1* allele against *P. aeruginosa*. Analysis of *npr-1* mutants confirmed these contrasting immune phenotypes for both avoidance behavior and nematode survival. Subsequent transcriptional profiling of *C. elegans* wildtype and *npr-1* mutant suggested that *npr-1* mediates defense against both pathogens through p38 MAPK signaling, insulin-like signaling, and C-type lectins. Importantly, increased defense towards *P. aeruginosa* seems to be additionally influenced through the induction of oxidative stress genes and activation of GATA transcription factors, while the repression of oxidative stress genes combined with activation of Ebox transcription factors appears to enhance susceptibility to *B. thuringiensis*.

**Conclusions:**

Our findings highlight the role of a single gene, *npr-1*, in fine-tuning nematode immune defense, showing the ability of the invertebrate immune system to produce highly specialized and potentially opposing immune responses via single regulatory genes.

**Electronic supplementary material:**

The online version of this article (doi:10.1186/s12864-016-2603-8) contains supplementary material, which is available to authorized users.

## Background

In contrast to higher vertebrates, which have adaptive immune response systems, invertebrates exclusively rely on the innate immune system (the immune system is here defined *sensu lato* as the organism’s defense against infection, including avoidance behavior, physical barriers, and physiological processes). For a long time it was assumed that only the adaptive system is capable of mounting highly specific defense responses. However, evidence is accumulating that invertebrates have surprisingly complex immune systems that in theory may have the potential to produce similar specificities [1–3]. Yet, to date, we possess only little information on the genetic and molecular mechanisms underlying such specificities. First insights into these mechanisms were previously obtained for the model nematode *C. elegans*, an important invertebrate system for studying immune defense [2, 4, 5]. For example, loss of function of the prolylhydroxylase encoding gene *egl-9* enhances susceptibility to *Staphylococcus aureus* [6] but resistance to *Pseudomonas aeruginosa* (PA01) [7] and *Bacillus thuringiensis* toxins [8]. Similarly, a loss of function of the Toll-like receptor gene *tol-1* increases susceptibility to *Salmonella enterica* but resistance to *Enterococcus faecalis* [9], even though the general importance of *tol-1* in worm immunity is unclear [5, 10].

Such specificities may not only be expressed by the nematode’s physiological immune system, but could also be expected for behavioral defenses. Such behaviors are a central component of immune defense *sensu lato* - next to protective barriers and physiological processes - and are likely to represent a highly economic immune defense strategy because they simultaneously reduce pathogen contact, and thus the risk of tissue damage, and also the necessity to activate the energetically costly physiological and cellular response [11]. *C. elegans* colonizes microbe-rich habitats in nature where it feeds on bacteria and yeasts [12–15]. Since these habitats also contain many pathogenic microorganisms, *C. elegans* has evolved distinct types of behavioral responses including physical avoidance, associative learning and reduced oral uptake of pathogens [4, 16–22].

Previous studies revealed the presence of substantial genetic variation among wild isolates of *C. elegans* in their behavioral response towards different pathogens [17, 19, 23–27]. In one case, namely the defense response against the Gram-negative bacterium *Pseudomonas aeruginosa*, this variation could be linked to the polymorphic neuropeptide receptor *npr-1* locus on the X chromosome*.* The gene *npr-1* was proposed to regulate *C. elegans’* immunity against PA14 either through controlling the aerotaxis response [17]*,* or through controlling both aerotaxis response and physiological immune defense [18]. *npr-1* is a homolog of the mammalian neuropeptide Y receptor gene and it is found in two different isoforms in *C. elegans* that result from a single amino acid change at position 215 (valine in isoform 215 V; phenylalanine in isoform 215 F) [28]. These isoforms do not only influence pathogen defense but also foraging behavior in response to oxygen concentrations [28, 29] and leaving behavior from lawns with the laboratory food bacterium *Escherichia coli* [30, 31].

The apparent complexity of the *C. elegans* defense against pathogens [1–3, 5] raises the question whether single pathways or genes can also fine-tune the behavioral defense response towards specific pathogens. To address this question we studied the genetic architecture of behavioral immune defense of *C. elegans* towards the Gram-positive pathogen *Bacillus thuringiensis*. This pathogen is likely to coexist with *C. elegans* in nature [15]. Some strains are nematocidal, whereby the host is infected by the oral uptake of spore-toxin mixtures. Infection of the gut is followed by toxin-mediated cellular damage of the intestinal epidermis, germination of spores and subsequent proliferation of vegetative cells, including expression of various virulence factors, ultimately resulting in nematode death [32–36]. Nematocidal *B. thuringiensis* induces pronounced behavioral responses in *C. elegans* [21, 23, 37, 38].

Here we explored genetic variation in *C. elegans* and used quantitative trait locus (QTL) analysis to characterize the genetic basis of behavioral immune defense against two pathogenic *B. thuringiensis* strains, whereby one strains (BT B-18679) is known to be more pathogenic than the other (BT B-18247) [39, 40]. Our QTL analysis was based on a panel of 200 recombinant inbred lines (RILs) and 90 introgression lines (ILs), derived from a cross between the *C. elegans* strains N2 and CB4856 [41, 42]. Our QTL analysis identified *npr-1* as one of the candidate genes, though with an opposite effect on avoidance behavior to that previously reported towards *P. aeruginosa* [17, 18]. Therefore, we further characterized the function of the *npr-1* gene in producing contrasting pathogen defense responses. Using *npr-1* mutants, we assessed the influence of the gene on both avoidance behavior and survival towards the two pathogen species, *B. thuringiensis* and *P. aeruginosa*. Moreover, we used RNAseq to identify differences in the pathogen-dependent transcription of *npr-1* down-stream targets. The functional importance of such differences was assessed through enrichment analysis of gene ontology (GO) categories, customized nematode-specific gene sets, which we collated from previous gene expression analyses, and transcription factor binding motifs.

## Results and discussion

### Two *C. elegans* wild-type strains differ in bacterial lawn leaving behavior

**Fig. 1:**
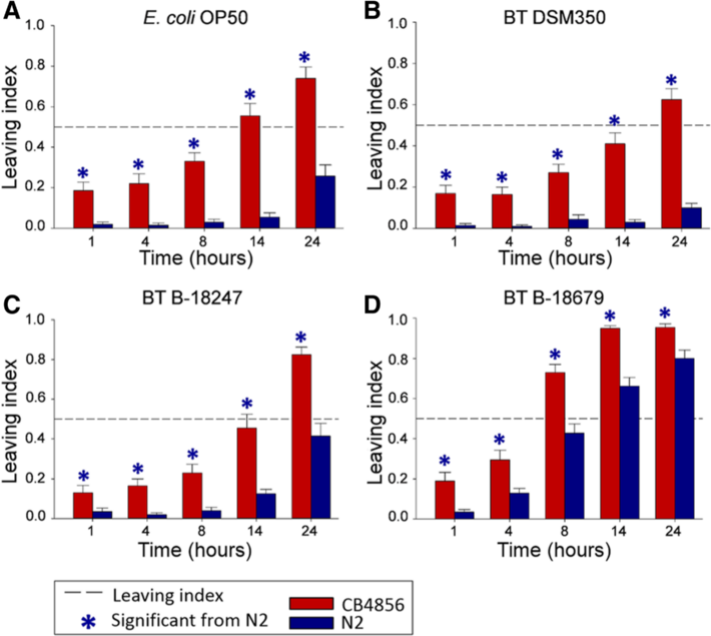
Variation in lawn leaving behavior between N2 and CB4856 towards different bacterial strains. Lawn leaving behavior towards: (**a**) the non-pathogenic *E. coli* strain OP50; (**b**) the non-nematocidal *B. thuringiensis* strain DSM350; (**c**) the nematocidal *B. thuringiensis* strain B-18247; and (**d**) the highly nematocidal *B. thuringiensis* strain B-18679. The Y axis shows the leaving index for N2 (*blue*) or CB4856 (*red*), calculated as the proportion of nematodes which left the bacterial lawn; the X axis indicates the different exposure time points. Asterisks denote the treatments for which leaving behavior of CB4856 is significantly different from N2. The dotted reference line shows a leaving index of 0.5. Error bars represent standard error of the mean. Raw data are given in sheet 1 of Additional file 2. Detailed statistical results are shown in Additional file 3

The standard laboratory strain N2 and the Hawaiian strain CB4856 showed significant variation in lawn-leaving behavior towards nematocidal *B. thuringiensis* strains (B-18679 & B-18247) and non-nematocidal strains (DSM350 & *E. coli* OP50). Lawn leaving served as a proxy for behavioral defense and was based on an assay (Additional file 1) related to those previously used to characterize *C. elegans* avoidance behavior [10, 16, 19, 21, 31, 37], in this case using peptone-free medium (PFM) to prevent *B. thuringiensis* spore germination outside the host (see Methods). Lawn leaving behavior was significantly higher for CB4856 compared to N2 on all tested bacterial strains and for all exposure time periods (Fig. 1; Sheet 1 in Additional files 2, 3 and 4). For both *C. elegans* strains, we observed a significant increase in leaving across time (Fig. 1). For both, the avoidance response towards the most pathogenic strain (B-18679) was higher than that towards the less pathogenic strain (B-18247) (Fig. 1c, d).

Our results confirm previously reported higher avoidance behavior and resistance of CB4856 compared to N2 towards one of the pathogens used in the current study, *B. thuringiensis* B-18247 [23]. Our findings are also consistent with two previous studies that demonstrated a higher OP50-patch leaving behavior [31] and a higher microsporidia resistance of CB4856 compared to N2 [43]. Interestingly, the opposite phenotype has been reported regarding the nematode’s response to two other pathogens, *P. aeruginosa* and *Serratia marcescens*. In these cases, N2 rather than CB4856 produced higher resistance and behavioral avoidance towards *P. aeruginosa* [17, 19], and higher avoidance towards *S. marcescens* [26]. Moreover, as the more pathogenic B-18679 was more strongly avoided than the less virulent B-18247 (Fig. 1c, d), *C. elegans* appears to be able to differentiate between different levels of pathogenicity of the same bacterial species. In this case, the difference in pathogenicity is likely due to expression of different Cry toxins that result in different infection patterns [37]. Based on our results we expect that the N2 and CB4856-derived RIL and IL populations are likely to contain sufficiently high levels of variation for a QTL analysis of avoidance behaviors towards the four chosen bacterial strains.

### Multiple QTLs and their interactions account for variation in avoidance behavior

We performed QTL analyses on *C. elegans* pathogen defense and revealed the genetic architecture of pathogen avoidance behavior to (i) be polygenic, (ii) include epistatically interacting loci, and (iii) incorporate general as well as pathogen-specific avoidance loci. In particular, our study simultaneously assessed the behavioral response of 200 RILs and 90 ILs [41, 42] towards four bacterial strains (two nematocidal *B. thuringiensis* and two non-nematocidal controls) at two exposure time points (14 h and 24 h) and with three replicates per treatment combination, using the same lawn leaving assays as above for N2 and CB4856. Below we present our results of a main-effect QTL analysis of the RIL population and an analysis of interaction effects among loci for the RIL population.

**Fig. 2:**
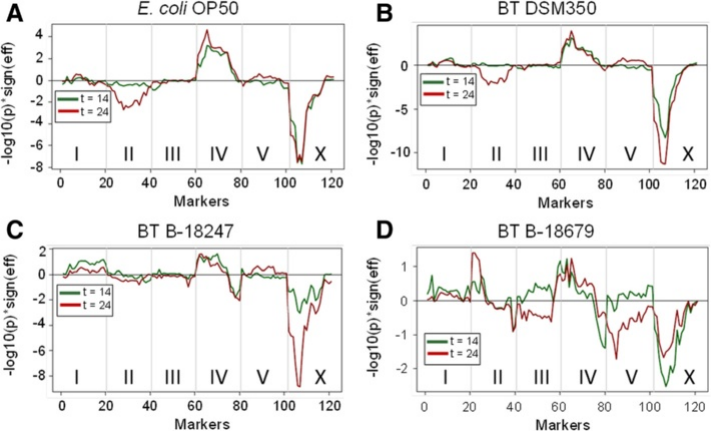
QTL profiles for single marker mapping of avoidance behavior. QTL analysis of avoidance of: (**a**) *E. coli* OP50; (**b**) the non-nematocidal *B. thuringiensis* DSM350; (**c**) nematocidal B-18247; and (**d**) highly nematocidal B-18679. The *X-axis* shows the markers along the five autosomes and the X chromosome. Vertical light gray lines denote the boundaries between chromosomes. The *Y-axis* indicates the association between the chromosomal markers and variation in avoidance. Significance is indicated by –log10 of the *p*-value obtained from the linear model, which is multiplied by the sign of the effect to indicate the N2 allelic effect on avoidance. A value above 0 indicates an increase in leaving caused by the N2 allele; a value below 0 indicates an increase in leaving caused by the CB4856 allele. By convention, values above +2 or below -2 are considered to indicate a significant influence. *Green* and *red* lines show the results after either 14 h or 24 h exposure, respectively

The main effect QTL analysis uncovered five main regions associated with avoidance: (i) one large region in the middle of chromosome II, for which the CB4856 allele(s) increase(s) leaving behavior of the non-pathogenic bacteria only at the 24 h time point (Fig. 2a, b); (ii) a region on chromosome II, for which the N2 allele(s) specifically increase(s) avoidance of the more pathogenic strain B-18679 at the 24 h time point (Fig. 2d); (iii) a region on the left arm of chromosome IV, for which the N2 allele(s) increase(s) leaving behavior towards controls and pathogens (Fig. 2a-d); (iv) a region on the right arm of chromosome IV, for which the CB4856 allele(s) increase(s) avoidance of pathogens at both time points (Fig. 2c, d); and (v) a region on chromosome X with the strongest effect on leaving behavior towards controls and pathogens (Fig. 2a-d), mediated by the CB4856 allele(s) and including a significant time effect on the response to the pathogenic bacteria (Fig. 2c, d). This very strong X chromosome effect was confirmed by the ILs (Additional file 5).

**Fig. 3:**
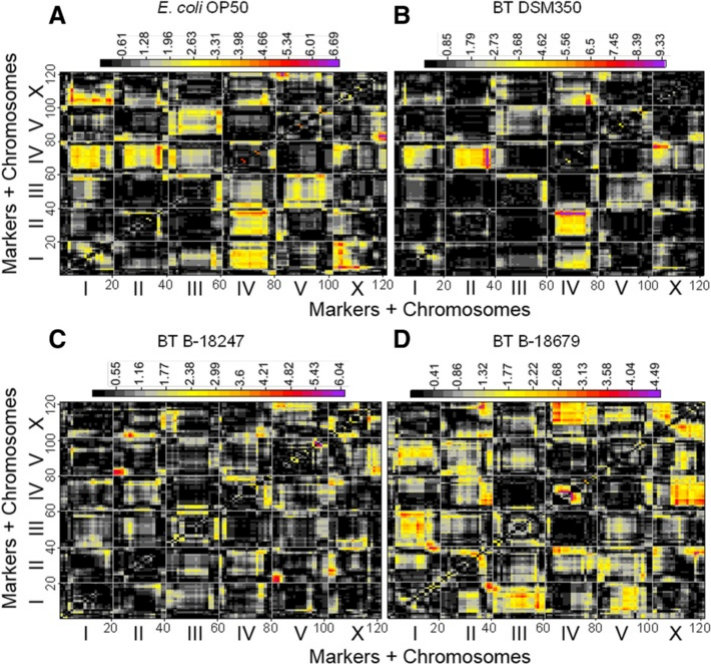
Heat-map of interaction effects for avoidance behavior. Results for avoidance of: (**a**) *E. coli* OP50; (**b**) DSM350; (**c**) nematocidal B-18247; and (**d**) nematocidal B-18679. The distribution of markers across the genome is shown on both axes. The chromosome boundaries are indicated by the thin vertical and horizontal lines. A color legend for significance of the interaction between markers is shown on top of the panels. Significance is in –log10(p). High significance values are given in “warm” colors with purple and red indicating the highest significances, followed by orange and then yellow. For example, in panel (**c**) showing the results for B-18247, the thin red area towards the bottom right indicates a significant interaction between loci from the beginning of the X chromosome (*horizonal axis*) and the middle of chromosome II (*horizontal axis*). The other slightly larger red area in the bottom right of this panel points to a significant interaction between the beginning of chromosome V (*horizontal axis*) and the beginning of chromosome II (*vertical axis*)

Our analysis of interaction effects among QTLs, using a standard interaction model (phenotype ~ time + marker1 * marker2), revealed several significant intra-genomic associations with an influence on lawn leaving behavior. For *E. coli*, significant interactions were found for at least two cases (Fig. 3a): (i) between the beginning of chromosome I and the first half of chromosome X; and (ii) between the end of chromosome II and almost the entire IV chromosome. For DSM350, we identified interaction effects between: (i) the end of chromosome IV and the first quarter of chromosome X; and (ii) the end of chromosome II and almost the entire IV chromosome (Fig. 3b). The latter seems to be specific for food patch leaving behavior as it was identified for both of the non-pathogenic bacteria (Fig. 3a, b). For the nematocidal B-18247, we found interaction effects at least between: (i) the beginnings of chromosome II and V; and (ii) the second quarter of chromosome II and the beginning of chromosome X (Fig. 3c). For the highly nematocidal B-18679, several interaction effects were identified including: (i) between the ends of chromosome I and II; and (ii) between the second quarter and the middle of chromosome IV (Fig. 3d). Interestingly, the X chromosome region with the strongest influence in the main effect model (Fig. 2) only contributed to very few significant interaction effects. One of these is an interaction with a chromosome I region,mediating avoidance of *E. coli* OP50 (Fig. 3a), and another one with a chromosome IV region, influencing avoidance of DSM350 (Fig. 3b).

Taken together, our results demonstrate that pathogen avoidance has a complex genetic architecture in *C. elegans*, which overlaps with, but differs from the response to non-pathogenic microbes. In particular, pathogen defense traits are related to the response to non-pathogenic bacteria, because they are affected by the same loci. Defense is thus in part determined by the general response to microbes, whereby pathogenicity of the bacteria may simply elevate the response mediated by a particular locus, as indicated for the X chromosome QTL (Fig. 2). Moreover, our results for the pathogen-specific QTLs are consistent with the previous finding that pathogen defense in invertebrate animals seems to rely on few loci and involve epistatic interactions among them [44, 45], possibly as a consequence of reciprocal coevolution among host and pathogens [44]. It will be a rewarding challenge for the future to characterize the genes underlying the pathogen-specific QTLs. Interestingly, the main effect QTL on the X chromosome was previously implicated in lawn leaving behavior with similar allelic effects towards the non-pathogenic *E. coli* OP50 (i.e., the CB4856 allele increases avoidance; [31]) but with opposite allelic effects towards *P. aeruginosa* (i.e., the N2 allele increases avoidance; [17–19]). In these cases, the QTL effect on chromosome X could be associated with variation in the gene *npr-1* [17–19, 31] and, at least towards *E. coli*, additionally the catecholamine receptor gene *tyra-3* [31].

### The *npr-1* gene affects defense against *B. thuringiensis*

The gene *npr-1* was previously linked to the strong-effect QTL on chromosome X [17], which we found in the current study to influence *C. elegans* defense towards *B. thuringiensis* in the opposite way than that towards *P. aeruginosa*. We therefore specifically tested whether this gene is indeed responsible for the contrasting phenotypes in pathogen avoidance and resistance.

We first studied the role of *npr-1* in lawn leaving behavior on *E. coli* and *B. thuringiensis* using two mutants (*npr-1(ad609)*, *npr-1(ur89)*), which were both previously shown to decrease pathogen avoidance behavior against *P. aeruginosa* [17, 18]. Another gene from the left arm of chromosome X was previously demonstrated to influence food patch leaving behavior, namely *tyra-3*, which encodes a tyramine receptor homologue [31]. Therefore, we further tested its involvement in pathogen defense with the knock-out mutant *tyra-3(ok325)*.

**Fig. 4:**
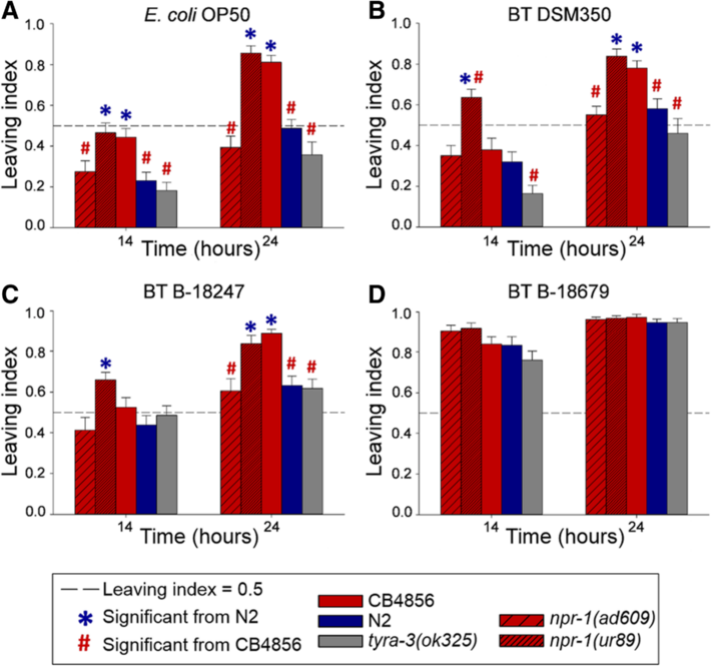
Lawn leaving behavior for N2, CB4856, and different mutants towards *E. coli* and *B. thuringiensis*. Results for avoidance of: (**a**) *E. coli* OP50; (**b**) non-nematocidal *B. thuringiensis* DSM350; (**c**) nematocidal *B. thuringiensis* B-18247; and (**d**) highly nematocidal B-18679. The *Y axis* shows the proportion of escaped worms (i.e., leaving index); the *X axis* shows the two time points at which leaving was scored. The asterisk (*) is shown for the treatments which differ significantly from N2 whereas the hash sign (#) indicates significant differences from CB4856. The dotted reference line shows a leaving proportion of 0.5 for orientation among sub-panels. Error bars represent standard error of the mean. Raw data are given in sheet 2 of Additional file 2; detailed statistical results in Additional file 6

**Fig. 5:**
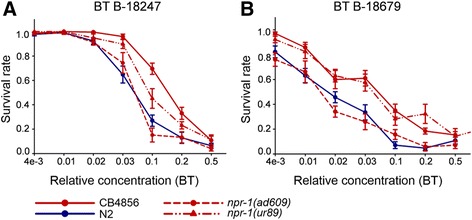
Survival of N2, CB4856, *npr-1(ad609)* and *npr-1(ur89)* on nematocidal *B. thuringiensis*. Results for: (**a**) nematocidal B-18247; and (**b**) highly nematocidal B-18679. Survival on the *Y axis* was plotted against relative *B. thuringiensis* concentration on the *X axis*. Error bars represent standard error of the mean. The raw data are provided in sheet 4 of Additional file 2; the statistical results in Additional file 8

Analysis of the two mutant *npr-1* alleles (*npr-1(ad609)*, *npr-1(ur89)*) yielded different results in avoidance and resistance (Figs. 4 and 5; sheets 2–4 of Additional files 2, 6, 7 and 8). In particular, avoidance behavior of *npr-1(ur89)* was similar to CB4856 but higher than that of N2 (Fig. 4, Additional file 6). In contrast, *npr-1(ad609)* always produced a low leaving rate, similar to N2 and clearly different from CB4856. On the highly pathogenic B-18679, avoidance behavior was extremely high at both time points without any significant differences among the *C. elegans* strains (Fig. 4d). In addition, the *tyra-3* mutant consistently showed a similar behavioral response to N2, irrespective of the bacterium and the exposure time (Fig. 4, Additional file 6).

The *npr-1* alleles produced similarly contrasting effects on survival rate, which is often used as a proxy for nematode immunity. For the RIL/IL parental strains, we found that CB4856 showed significantly higher resistance than N2 on both nematocidal *B. thuringiensis* strains (Fig. 5; Additional files 7, 8 and 9). Moreover, the *npr-1(ur89)* mutant was significantly more resistant than N2 and as resistant as CB4856 on both nematocidal pathogens, whereas *npr-1(ad609)* was as susceptible as N2 on both pathogenic strains (Fig. 5, Additional file 8). None of the *C. elegans* strains showed any mortality under control conditions (results not shown).

We conclude that the two mutant *npr-1* alleles produce opposite effects on both behavioral avoidance of the four bacterial strains and also resistance against nematocidal *B. thuringiensis*. Consequently, variation in *npr-1* may only partially explain the strong main effect QTL on the X chromosome. The difference between the two *npr-1* alleles in avoidance and resistance of *B. thuringiensis* is surprising, because both alleles behaved similarly in previous studies investigating resistance against *P. aeruginosa* [17, 18]. Yet the two alleles carry different mutations: *npr-1(ad609)* two in exons 2 and 3, whereas the mutation of *npr-1(ur89)* falls into exon 3 (http://www.wormbase.org). The exact reasons for the different effects of these alleles clearly deserve further investigation in the future, ideally including additional loss-of-function and also reduced-function *npr-1* alleles in combination with a tissue-specific analysis of the mutational effects. We further conclude that the *tyra-3* gene does not appear to influence the assayed phenotypes (Figs. 4 and 5), including avoidance of *E. coli* OP50, which was however previously demonstrated in a separate study [31]. The difference in results could be due to variation in experimental approaches. For example, we directly characterized leaving behavior, whereas the previous study scored activity as a proxy for leaving behavior [31].

### Contrasting effect of *npr-1* on defense against *Pseudomonas aeruginosa*

**Fig. 6:**
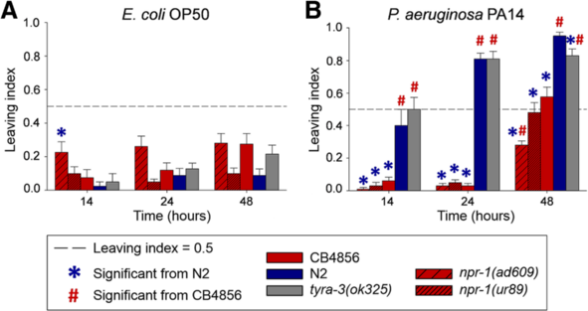
Lawn leaving behavior for N2, CB4856 and different mutants towards *E. coli* and *P. aeruginosa*. Results for: (**a**) *E. coli* OP50; and (**b**) *P. aeruginosa* strain PA14. The *Y axis* shows the proportion of escaped worms; the *X axis* shows the three time points at which leaving was scored (12 h, 24 h, and 48 h). The asterisk (*) denotes the treatments which differ significantly from N2, whereas the hash sign (#) indicates significant differences from CB4856. The dotted reference line shows a leaving proportion of 0.5. Error bars represent standard error of the mean. The raw data are provided in sheet 6 of Additional file 2; detailed statistical results in Additional file 12

We sought to confirm the previously published finding [17–19] that the wild-type N2 produces higher resistance and stronger avoidance behavior towards the pathogen *P. aeruginosa* PA14 than the Hawaiian strain CB4856 and two *npr-1* mutants [17–19], thus contrasting with our above results for *B. thuringiensis*. Here, we specifically re-evaluated these previous results under our laboratory conditions and assay protocols, using the peptone-rich NGM plates required for expression of *P. aeruginosa* virulence. We first used the lawn leaving assay to assess the avoidance response against PA14 at different exposure time points. Consistent with previous findings, the *npr-1* mutants and CB4856 showed significantly lower PA14 pathogen avoidance than N2 across all time points (Fig. 6; sheets 5 and 6 of Additional files 2, 10, 11 and 12). For the 48 h time point the mutant *npr-1(ad609)* even had a lower leaving response than CB4856 (Fig. 6b). On OP50, leaving behavior was similar for all *C. elegans* strains at all time points except at time point 14 h, when the mutant *npr-1(ad609)* showed a more pronounced leaving behavior than N2 (Fig. 6a, Additional file 12). In this assay, we also included a *tyra-3* mutant, which expressed a similar leaving response to N2 under all conditions except at time point 48 h, where its leaving response against PA14 was reduced in comparison to N2, but still significantly higher than that of the remaining strains (Fig. 6b, Additional file 12).

**Fig. 7:**
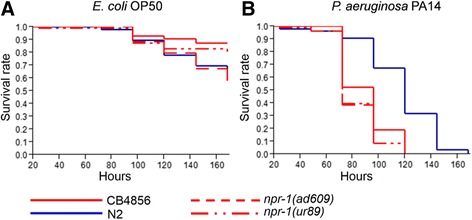
Survival of N2, CB4856, *npr-1(ad609)* and *npr-1(ur89)* on *E. coli* and *P. aeruginosa*. Results for: (**a**) *E. coli* OP50; and (**b**) *P. aeruginosa* strain PA14. Survival (*Y axis*) was plotted against time in hours (*X axis*). We used the Kaplan-Meier method to calculate survival fractions. The raw data are given in sheet 7 of Additional file 2; statistical results in Additional files 13 and 14

We next evaluated the effect of *npr-1* on resistance against PA14 using standard *C. elegans* survival assays. All strains survived less on the pathogen PA14 than on the control OP50 (Fig. 7; sheet 7 of Additional files 2 and 13). On the pathogen, N2 was significantly more resistant than all other tested strains (Fig. 7b, Additional file 14).

We conclude that *npr-1* directly influences avoidance of PA14, in agreement with previous work [17–19]. In these studies, *npr-1* was suggested to affect PA14 resistance either as a consequence of hyperoxia avoidance behavior only (proposed by Reddy et al., [17]) or through both hyperoxia avoidance and the regulation of physiological immune responses (proposed by Styer et al., [18]).

Our results further demonstrate that the two *C. elegans* wild-type strains express opposite phenotypes on the two tested pathogens and that this contrast may be mediated at least partially by *npr-1*, as one of the *npr-1* alleles also produces an opposite phenotype relative to N2. Such opposite effects in the wild-type strains indicate specific interactions with pathogens. The underlying genetics for such specificities have not yet been explored for behavioral immune defense. Some information is available for physiological and cellular immune specificities. In the higher vertebrates, such specificities can be mediated by components of the adaptive immune system such as the highly variable receptors of the major histocompatibility complex or the highly variable T and B cell receptors. Similar specificities have also been recorded in invertebrates [1, 46], where they may be due to different immune signaling cascades. For example, in *Drosophila*, the immune deficiency pathway appears to be more important in the systemic response to Gram-negative bacteria, whereas the Toll pathway is more important towards Gram-positive bacteria and fungi [47]. Moreover, in *C. elegans*, mutations in the *egl-9* and *tol-1* gene enhance resistance against some pathogens, while simultaneously increasing susceptibility to other pathogens (see introduction and [5–8, 10]). Our study thus provides one of the few examples which demonstrate that a single gene, in this case the neuropetide Y receptor homolog gene *npr-1*, produces contrasting pathogen specificities in an invertebrate.

At the same time, it is less clear how exactly *npr-1* causes these contrasting phenotypes. Previous work on nematode social behavior demonstrated that *npr-1* influences worm aggregation, lawn bordering and clumping through its effect on aerotaxis behavior. The two tested *npr-1* alleles and also that of the CB4856 strain result in a preference towards lower oxygen concentrations usually found at the edge of the bacterial lawn [48, 49], whereas the N2 allele shows no such preference. A similar difference in aerotaxis behavior may also explain the reduced *P. aeruginosa* avoidance and resulting higher susceptibilities of the CB4856 and *npr-1* mutant strains, which remain in longer contact with the harmful pathogen, because the bacterial lawn boundaries show the preferred lower oxygen concentrations [17]. An involvement of such aerotaxis behavior in the *B. thuringiensis* response of CB4856 and one of the *npr-1* mutants would then require that the preferred oxygen concentration is outside of the bacterial lawn, which is unlikely to be the case (assuming higher oxygen concentrations outside, where no oxygen is consumed by proliferating bacteria). Thus, it is conceivable that CB4856 and the one *npr-1* mutant are directly responding to a compound produced by *B. thuringiensis*, and that this response is less pronounced in the N2 strain and the other *npr-1* mutant. In this context, it is worth noting that the high variation between N2 and CB4856 in their leaving response towards the control *E. coli* OP50 was only observed on peptone free PFM but not the peptone-rich NGM assay plates (Figs. 1a and 4a versus Figs. 6a and Additional file 10). This is most likely explained by bacterial proliferation, which is possible on NGM but not PFM assay plates. In turn, the lack of proliferation on the PFM plates is unlikely to coincide with large variation in oxygen concentration, such that an aerotaxis response should be less pronounced under these conditions. Yet, a non-proliferating, static bacterial population may produce particular metabolites, which then could have induced the CB4856 avoidance response.

### *npr-1* influences the transcriptomic response to *B. thuringiensis* and *P. aeruginosa*

**Fig. 8:**
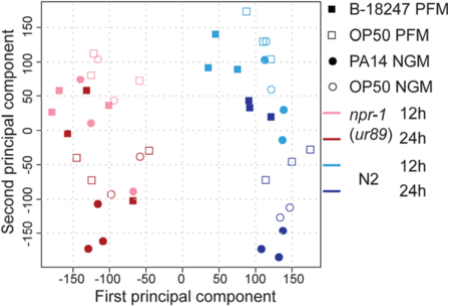
Principal component analysis of transcriptomic variation. Variation is assessed for *npr-1(ur89)* and N2 upon exposure to pathogens (B-18247 and PA14) and the control bacterium OP50. N2 is shown in blue whereas *npr-1(ur89)* is shown in red. *Light* and *dark* colors indicate the early and late time point, respectively (12 h versus 24 h). Filled and open symbols denote exposure to pathogens (B-18247 and PA14) and control bacteria (OP50), respectively. NGM is the nematode growth medium enriched with peptone for the PA14 assay plates, PFM refers to peptone-free NGM used for the B-18247 assay plates

To explore the mechanisms underlying the *npr-1* mediated contrasting effects on immune defense, we assessed whether *npr-1* differentially affects gene expression in the presence of either of the two pathogens. Using RNAseq we compared the transcriptomes of the N2 and *npr-1(ur89)* strains exposed to either the nematocidal *B. thuringiensis* B-18247, the pathogenic *P. aeruginosa* PA14, or the control *E. coli* OP50. We chose the mutant *npr-1(ur89)* because it showed differential leaving behavior and survival on both pathogens compared to N2 (Figs. 4 and 6). Exposure experiments were performed on Agar plates fully covered with bacterial lawns, thus reducing possible avoidance behaviors and producing comparable levels of lawn occupancy for the worms from the various treatment combinations. RNA transcript levels were characterized at two time points, 12 h and 24 h of pathogen exposure. We used principal component analysis (PCA) to explore which experimental factors generated different transcriptional responses. The first principal component indicated that the two nematode strains vary in their transcriptional signature to all three bacteria (Fig. 8). The second principal component highlights variation across several additional factors. The strongest effect stems from exposure time (light versus dark colors; Fig. 8). Additional influences can be seen for pathogen exposure versus the corresponding control, especially at the later time point (filled versus open symbols of the same type; Fig. 8) and also a clearly distinct signal after 24 h exposure to PA14 compared to all other conditions (filled dark colored circles towards the bottom of the graph; Fig. 8). These latter differences are more pronounced for N2 than the *npr-1* mutant, especially as N2 produces clearly distinct treatment signatures at the later 24 h time point (i.e., clearly separated dark blue open and filled circles and squares; Fig. 8). One possible reason for lower differentiation in the *npr-1* mutant may be a lower number of differentially expressed genes compared to the N2 strain. This was indeed the case, especially upon pathogen exposure (Table 1), suggesting that mutations in *npr-1* somehow compromise the signalling response to pathogen infection.

**Table 1:** Number of up- and down-regulated genes in the N2 and *npr-1(ur89)* strains

	B-18247		PA14	
*C. elegans*	12 h	24 h	12 h	24 h
Up-regulated				
N2	1393	1094	222	529
* npr-1(ur89)*	179	191	188	352
Down-regulated				
N2	2384	458	81	1233
* npr-1(ur89)*	117	70	58	370

RNAseq was used to assess variation in gene expression among the *C. elegans* N2 and *npr-1(ur89)* mutant strain in response to exposure to nematocidal *B. thuringiensis* B-18247 and *P. aeruginosa* PA14, always relative to the respective *E. coli* OP50 control. Gene expression variation was studied at two time points, 12 h and 24 h after initial exposure. The results are shown separately for the up- and down-regulated genes (top and bottom part of the table, respectively)

**Fig. 9:**
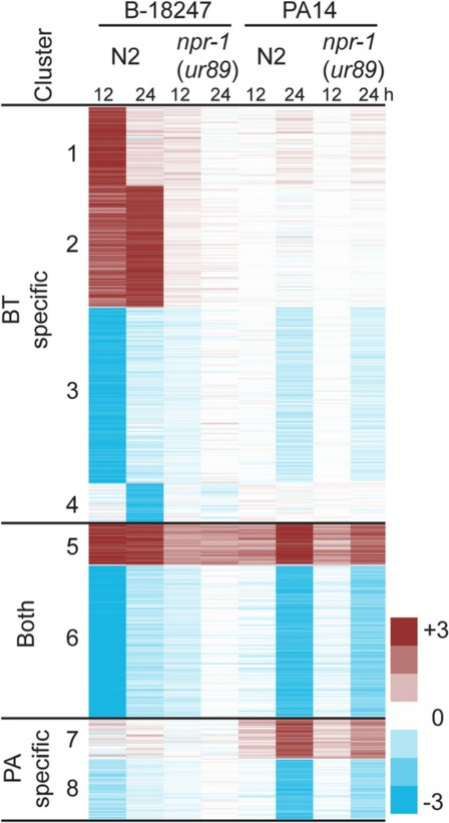
Co-regulation of the differentially expressed genes. K-means cluster analysis yielded eight clusters of co-regulated genes, as indicated by the numbers on the left. The *top* of the graph shows the different treatment conditions, including *C. elegans* strain, pathogen strain and exposure time point. *Red* refers to up-regulated genes, whereas blue to down-regulated genes, always upon pathogen exposure relative to the corresponding *E. coli* OP50 control. High color intensities indicate strong expression differences to the control (see legend in the *bottom right* corner). The complete results are given in Additional file 15

To identify groups of co-regulated genes, we next performed K-means clustering on the significant gene sets. The resulting eight clusters confirm that the transcriptional response is influenced by the *C. elegans* strain, the pathogen strain and also the exposure time point (Fig. 9; Additional file 15). In detail, clusters 1, 2, 3, and 4 refer to genes with strong differential expression upon exposure to only pathogenic *B. thuringiensis* B-18247 in only the *C. elegans* N2 strain, but neither the *npr-1(ur89)* mutant on the same pathogen nor any of the other treatments with *P. aeruginosa* (e.g., the stronger the color intensity in Fig. 9, the stronger the expression difference between pathogen versus non-pathogen exposure). This result again highlights that the *npr-1* mutant shows generally lower responsiveness in inducible gene expression (i.e., most clusters do not show high color intensity in Fig. 9). Clusters 1, 2, 3 and 4 only responded to the pathogen B-18247, and clusters 7 and 8 only or at least predominantly to PA14. Two clusters are specific to expression variation at the 12 h time point, in both cases upon exposure to B-18247 (i.e., clusters 1 and 3), whereas four clusters indicate a more pronounced response at the later 24 h time point, either towards only B-18247 (clusters 2 and 4) or only PA14 (clusters 7 and 8). The remaining two clusters highlight patterns of early or continuous transcriptional response towards B-18247 and late transcriptional response towards PA14 (clusters 5 and 6; Fig. 9). None of the identified clusters showed an opposite gene expression pattern between either N2 and the *npr-1* mutant (e.g. up in N2 and down in the *npr-1* mutant) or the two pathogens (e.g. up after *B. thuringiensis* but down after *P. aeruginosa* exposure). Taken together, clusters 5 and 6 appear to encompass a general defense response against both pathogens, whereas the clusters 1, 2, 3, and 4 define the specific response to B-18247 and clusters 7 and 8 that to PA14. Therefore, the latter two groupings are likely to account for the observed *npr-1* dependent defense differences towards the two pathogens. We thus conclude that the considered mutation in the *npr-1* gene causes a decreased transcriptomic response to the two pathogens, which induce overlapping and distinct sets of differentially expressed genes.

### Different functions and signaling processes are affected by the pathogen-dependent *npr-1*-specific transcriptome

**Fig. 10:**
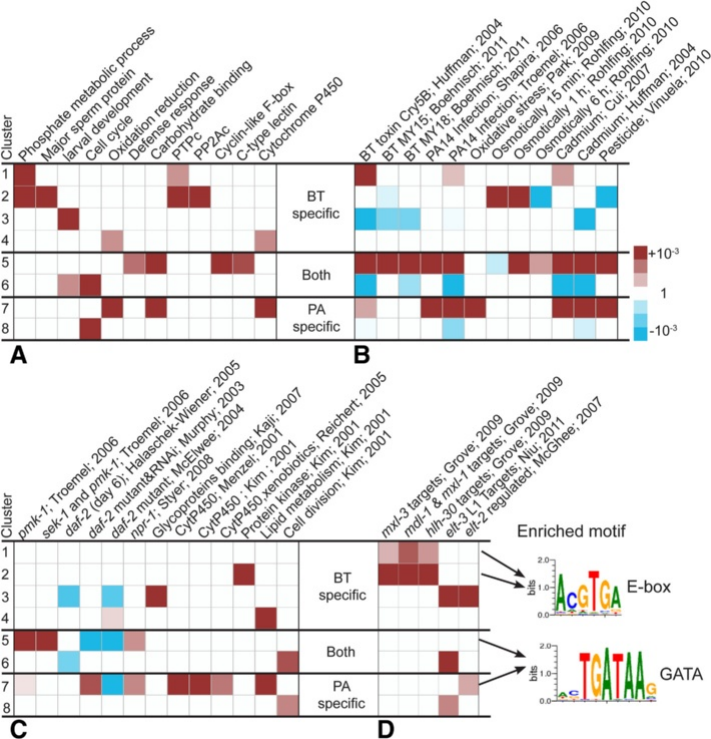
Functional consequences of gene expression variation between *npr-1(ur89)* and N2 upon pathogen exposure. **a** Enrichment of gene ontology (GO) terms. The shown terms were significant with FDR < 0.05 (Additional file 17). **b** Overview of enrichment of pathogen- and stress-induced gene sets, inferred from EASE analysis on the various clusters of co-regulated genes. The significantly enriched gene sets are indicated on the top and include - from *left* to *right* - differentially expressed genes upon exposure to (i) *B. thuringiensis* Cry5B toxin [89]; (ii)-(iii) the same B-18247 strain used in the current study in *C. elegans* isolate MY15 or MY18 [90]; (iv)-(v) the same PA14 strain used here [66, 91]; (vi) Oxidative stress response [92]; (vii)-(ix) Osmotic induction [93]; (x)-(xi) Heavy metal Cadmium dysregulated genes [89, 94]; (xii) Pesticide influence [58]. **c** Overview of enriched gene sets for selected immunity pathways and general categories, including (i)-(ii) the p38 MAPK pathway (*pmk-1* and *sek-1* targets; [91]); (iii)-(v) insulin signalling (*daf-2* targets; [95–97]); (vi) *npr-1* targets [18]; (vii) Glycoproteins; (viii)-(x) Cytochrome P450 [98–100]; (xi) Protein kinase [98]; (xii) Lipid metabolism [98]; (xiii) Cell division [98]. **d** Enrichment of Ebox and GATA motifs and their transcriptional targets. The enriched gene sets were inferred with EASE and are indicated at the top, including differentially expressed genes in mutants of (i)-(iii) E-box transcriptions factors [101] or (iv)-(v) GATA transcription factors ELT-2 and ELT-3 [102, 103]. Enriched transcription factor binding motifs were inferred with AMD and are shown on the right (Additional file 17). In all panels, the clusters are given on the very left and are identical to those in Fig. 9. *Red* color indicates an enrichment for up-regulated genes per gene set, blue that for down-regulated genes per gene set. Color intensity corresponds to the significance level, inferred by EASE analysis (see scale at the *right side*)

**Fig. 11:**
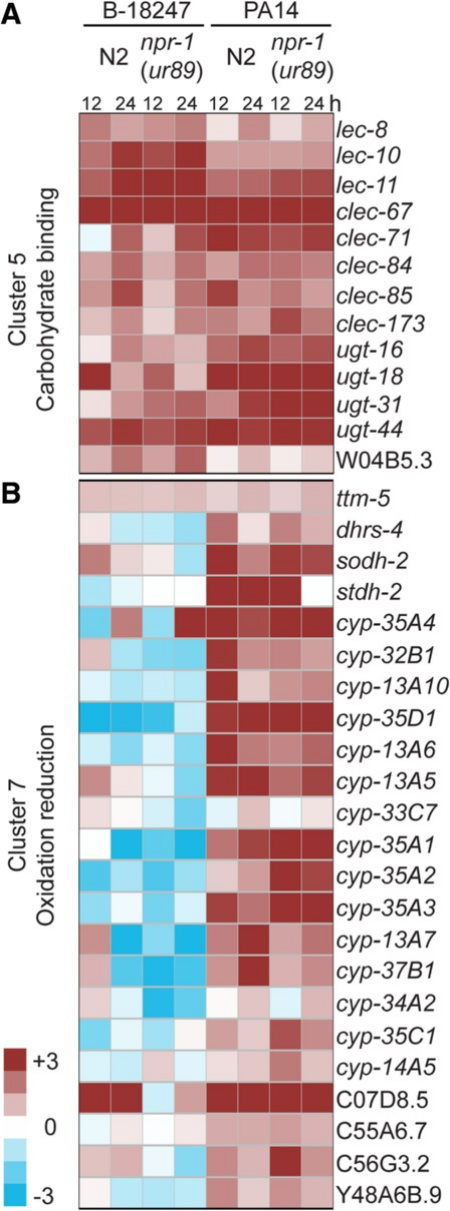
Example for enriched Gene ontology terms. **a** Heatmap for carbohydrate binding on cluster 5; **b** for oxidation reduction on cluster 7. *Red* and *blue* color indicate up- and down- regulation compared to OP50

We used enrichment analysis as a statistical tool to explore the possible functions of the differentially regulated gene clusters. Four types of enrichment analyses were performed, which aim to identify significant over-representation of (i) genes with a specific gene ontology (GO) term (GO term analysis); (ii) customized nematode-specific gene sets, inferred from previous gene expression analyses and based on the program EASE (EASE analysis); (iii) genes with specific transcription factor-binding motifs (Motif analysis), and (iv) expression QTLs (eQTLs). The customized enrichment analysis with the program EASE [50] was based on a large database of all previous *C. elegans* transcriptome studies, WormExp [51], which we collated from published work. These studies investigated differential gene expression (i) across development, (ii) in specific tissues, (iii) in worms with defined mutations or subjected to RNAi-knockdown of specific genes, or (iv) upon exposure to environmental stimuli such as pathogens, heavy metals, and other chemical compounds [51]. The GO term and Motif analyses were based on published methods, such as DAVID [52, 53] and AMD [54]. Analysis of eQTL enrichment expression differences, using the eQTL database collated from different previous eQTL analyses, all based on RIL panels derived from N2 and CB4856 as parental lines [41, 55–60], thus potentially allowing us to link the identified QTLs to the expression variation inferred against pathogens. The results are summarized in Figs. 10 and 11, Table 2, Additional files 16 and 17, and explained in more detail below.

**Table 2:** eQTL enrichment analysis on identified expression clusters

Expression cluster	Location of eQTL		eQTL set
	Chromosome	Approximate position	
1	V	3.7 M	Rockman
2	IV	6.6 M	Viñuela (old)
3	X	15.5 M	Rockman
4	-	-	-
5	X	2.3 M	Rockman
	X	5.8 M	Rockman
	X	10.9 M	Rockman
	IV	6.3 M	Rockman
	III	1.9 M	Rockman
6	I	5.0 M	Viñuela (juvenile)
7	X	15.7 M	Rockman
	V	12.3 M	Rockman
8	I	3.9 M	Rockman

eQTL enrichment analysis was performed to identify significant overlaps between the genes underlying a specific cluster in our analysis (first column; see also Fig. 9) and previously characterized gene sets that define particular eQTLs (last column). Such overlaps can then be linked to the specific QTL regions within the genome (second and third column), which may then contain regulatory elements important for the expression variation in our study and which may also have been identified as QTLs for the observed variation in behavioral immune defense (Figs. 2 and 3). For further details see Additional file 16. No significant enrichment was found for cluster 4

We first focus on the general defense response against the two pathogens, which is defined by two clusters (i.e, clusters 5 and 6; Fig. 9). These clusters are enriched for genes previously implicated in *C. elegans* pathogen defense. These include genes involved in carbohydrate binding (Fig. 10a), most likely mediated by C type lectin-like genes, many of which underlie this GO term (Additional file 17 under GO) and which are up-regulated across treatment conditions (Fig. 11a) and have repeatedly been implicated in *C. elegans* immunity, possibly as pathogen recognition receptors or antimicrobials [11, 61–64]. These two clusters are also enriched for genes which were previously shown to respond to exposure to the same pathogens and other types of stressors, such as heavy metals, osmotic stress, or pesticides (Fig. 10b). The upregulated genes appear to be controlled by two of the main *C. elegans* immunity signalling cascades, the p38 MAPK and the insulin-like receptor pathways (Fig. 10c) [2], and also the *npr-1* gene (Fig. 10c) [18]. They also show an enrichment in their promotor sequences for a GATA binding motif, although not for known targets of the GATA transcription factors ELT-2 and ELT-3 (Fig. 10d). They are also enriched for gene sets defined by eQTLs on chromosome I, III, the middle of chromosome IV, and the left arm and the middle of chromosome X (Table 2). One of the enriched X chromosome eQTLs encompasses the *npr-1* gene, another the gene *sek-1* of the p38 MAPK cascade, and the one on chromosome IV may include the MAPK gene *jnk-1* or the p38 homolog *pmk-1*, additionally supporting the role of these genes in the nematode’s expression response. The enriched eQTLs from chromosome IV and the left arm of the X chromosome also lie within the QTLs identified to influence behavioral defense against *B. thuringiensis* (Fig. 2). Taken together, we conclude that clusters 5 and 6 comprise the components of a general defense response, apparently active not only against pathogens but also other stressors and mediated by central stress and immune response pathways. In the *npr-1* mutant, this defense response is strongly reduced towards both pathogens.

**Fig. 12:**
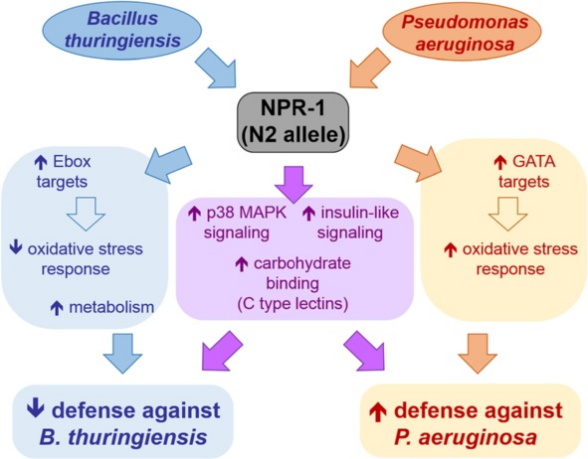
Model of *npr-1* dependent effects on pathogen defense in the N2 *C. elegans* strain. Exposure to both pathogens leads to an *npr-1* dependent activation of carbohydrate-binding factors, such as C-type lectin-like proteins, and also two central immune signaling cascades, the p38 MAPK and the insulin-like pathways, which could all enhance pathogen resistance (*middle* part of the graph). Upon exposure to *P. aeruginosa* PA14 (*right side*), *npr-1* also influences the activation of a general stress response, via one or several GATA transcription factors(s), which increases oxidative stress resistance and thus resistance to the pathogen. The response to *B. thuringiensis* (*left side*) is mediated by *npr-1* through one or several Ebox transcription factors, resulting in a reduced oxidative stress response and increased metabolic activity as a possible cause of enhanced pathogen susceptibility. *Arrows* with light colors indicate uncertain connections

The specific response to *P. aeruginosa* is captured by two clusters (i.e., clusters 7 and 8; Fig. 9). They are enriched for eQTLs on chromosome I, V, and X (Table 2), although in chromosomal regions without any known regulator of immune defense. These clusters, especially the upregulated cluster, are however enriched for genes previously shown to respond to infection by the same pathogen (Fig. 10b) and also those involved in the response to oxidative stress and xenobiotics, including cadmium and pesticides (Fig. 10a, b). The response to oxidative stress and xenobiotics is dominated by an up-regulation of cytochrome P450 genes (Fig. 11b; Additional file 17). The up-regulated cluster is further influenced by the two main immunity pathways, the *npr-1* gene, and also a GATA transcription factor (Figs. 10c, d). All of the latter components have previously been shown to be central for immune defense against *P. aeruginosa*, especially the p38 MAPK signalling cascade [65] and the GATA transcription factor ELT-2 [66]. The transcriptomic response to *P. aeruginosa* infection is additionally strongly influenced by cytochrome P450 expression, possibly as part of a general stress response to reduce oxidative stress (Fig. 10c). Because these responses are activated more strongly in the N2 strain, they are likely to mediate the observed higher resistance and behavioral defense for this strain compared to the *npr-1(ur89)* mutant (Figs. 6 and 7 and model in Fig. 12).

The specific response to *B. thuringiensis* is defined by four clusters, two up-regulated and two down-regulated groups of genes (i.e., clusters 1–4; Fig. 9). They are enriched for eQTLs in the middle of chromosome IV (Table 2), which could contain known defense regulators against *B. thuringiensis*, the MAPK genes *jnk-1* and *pmk-1* [67], and which lies within the QTL above identified to contribute to behavioral defense against this pathogen (Fig. 2). Enriched eQTLs are additionally found on the left arm of chromosome V and the right arm of the X chromosome (Table 2), in both cases without a link to any known immune regulator. Moreover, the upregulated clusters show an over-representation of genes involved in metabolic processes and phosphatase activity (Fig. 10a). They also include genes known to respond to the same pathogen and cadmium, as well as genes that are usually down- rather than up-regulated upon osmotic and pesticide stress (Fig. 10b). These two upregulated clusters are enriched for an Ebox transcription factor binding motif and the corresponding targets (Fig. 10d). The two down-regulated clusters show an enrichment for oxidation reduction and developmental genes (Fig. 10a), genes responsive to *B. thuringiensis* and cadmium (Fig. 10b), and genes controlled by insulin-like signalling, including glycoproteins (Fig. 10c). One of the down-regulated clusters also shows an enrichment of the GATA transcription factor targets (Fig. 10d).

Taken together, the results of our enrichment analyses allow us to propose a possible mechanistic basis for the contrasting defense effects of the *npr-1* gene. It is worth reiterating that N2 and the mutant only differ in the presence of a mutation in the *npr-1* gene. Therefore, the differences observed between strains must be influenced by the allelic variation in this gene. The higher resistance and avoidance behavior of N2 towards *P. aeruginosa* is likely influenced by the activation of GATA and/or p38 MAPK targets and/or the induced oxidative stress response (see above; Fig. 12). The situation is less clear for the response to *B. thuringiensis*. Because N2 produces lower defenses against *B. thuringiensis* than the *npr-1(ur89)* mutant (Figs. 4 and 5), and because any differential gene expression is repressed in the *npr-1(ur89)* mutant (Table 1; Fig. 9), the specific activation of certain genes in N2 (i.e., for the two up-regulated clusters 1 and 2) and/or the suppression of other gene groups in N2 (i.e., the down-regulated clusters 3 and 4) must account for the observed lower resistance and avoidance response against this pathogen. We hypothesize that this may possibly be mediated by one of the following two processes or a combination thereof (see model in Fig. 12): (i) the lower oxidative stress response in N2 could lead to increased susceptibility towards *B. thuringiensis*, in analogy to the effect recently described towards *E. faecalis* [68] and assuming that *B. thuringiensis* toxicity causes oxidative stress (which is however currently unknown); and/or (ii) an activation of metabolic processes could be disadvantageous during pathogen infection, because it may reduce availability of energetic resources that can be invested in immune defense and because metabolic products may be exploited as a source of nutrition by the pathogen. Any of the other implicated functions (Fig. 10) may also contribute to enhanced susceptibility in an as yet unknown manner. These processes then seem to be influenced by *npr-1* through Ebox-specific transcription factors. Interestingly, the higher susceptibility is apparently caused by an activation of specific functions and signalling processes rather than their absence or at least reduced activity. This may indicate a sub-optimal response to this specific pathogen in the N2 strain or represent a consequence of pathogen-mediated manipulation of host responses, which are widespread among pathogens [69] and which have also been shown for another *Bacillus* species, *Bacillus nematocida*, to change *C. elegans* behavior and intestinal responses [70]. At the moment, it is unclear in what way the indicated processes influence either behavioral or physiological responses or both simultaneously. This represents a challenging topic for future research.

## Conclusion

Our study revealed a complex genetic architecture comprising several epistatically interacting QTLs associated with variation in *C. elegans* pathogen avoidance behavior. The most significant QTL encompassed the gene *npr-1*. Our functional analyses of this gene revealed a contrasting effect of *npr-1* on *C. elegans* immune defense, as assessed through both behavioral and also survival phenotypes. In particular, the CB4856 allele was associated with faster lawn leaving behavior and higher survival than the N2 allele on *B. thuringiensis*, whereas it was associated with lower lawn leaving behavior and lower survival on *P. aeruginosa*. A further characterization of the exact role of *npr-1* suggested that it mediates differential regulation of defense genes via either GATA transcription factors leading to increased immune defense towards *P. aeruginosa* or Ebox transcription factors leading to decreased immune defense towards *B. thuringiensis*. Our study thus demonstrates that a single gene in *C. elegans* mediates contrasting pathogen-specific defense responses.

## Methods

### *C. elegans* and bacterial strains

*C. elegans* strains: (i) the standard wild-type strains N2 and CB4856; (ii) 200 Recombinant Inbred Lines (RILs) and 90 Introgression lines (ILs) generated from crosses between N2 and CB4856 [41, 42, 55, 60]; and (iii) two distinct mutant alleles of *npr-1*, *npr-1(ur89)* X (strain IM222) and *npr-1(ad609)* X (strain DA609), and also the *tyra-3* knock-out allele *tyra-3(ok325)* X (strain VC125). The three mutant strains were obtained from the Caenorhabditis Genetics Center (CGC; http://www.cbs.umn.edu/CGC/) and were all generated in the N2 background. All worm strains were maintained at 20 °C on Nematode Growth Medium (NGM) plates with the non-pathogenic *E. coli* OP50 as an *ad libitum* food according to standard protocols [71]. All mutants were backcrossed at least three times to N2. Presence of the target mutation was confirmed for the two *npr-1* mutants by sequencing the *npr-1* gene at the location of the mutations and for the knock-out mutant *tyra-3* by polymerase chain reaction (PCR) analysis of the deleted region.

Bacterial strains: (i) two nematocidal strains of *B. thuringiensis*, NRRL B-18247 and NRRL B-18679, originally provided by the Agriculture Service Patent Culture Collection (United States Department of Agriculture, Peoria, Illinois, USA); (ii) the non-nematocidal *B. thuringiensis* strain DSM350, originally obtained from the German Collection of Microorganisms and Cell Cultures (Deutsche Sammlung von Mikroorganismen und Zellkulturen GmbH, DSMZ, Braunschweig, Germany); (iii) the pathogenic *P. aeruginosa* strain PA14, obtained from Dennis H. Kim, Boston, USA; and (iv) the non-pathogenic *E. coli* OP50. Before the start of this study, the three *B. thuringiensis* strains were cultured in large quantities as previously described [32]. The cultures consisted mainly of spores associated to nematocidal toxins in the case of B-18679 and B-18247, and non nematocidal toxins in the case of the control DSM 350. All cultures were set to a concentration of 1.5 × 10^10^ particles/ml, assessed through standard Thoma counting chambers and microscopic analysis. The cultures were cryo-preserved in aliquots at –20 °C; spore viability and pathogenicity are not affected by freezing under these conditions [38, 40, 72]. Usage of viable spore aliquots from the same starting culture allowed us to minimize variation across experiments, thus enhancing comparability of the data from different study approaches. In all cases, aliquots were thawed directly before each experiment. The bacterial cultures were then diluted, as indicated below in the description of the various assays.

### Lawn leaving assays

The assay was designed for 9 cm petri dishes with either Peptone free NGM (PFM) for *B. thuringiensis* assays or peptone-rich NGM for PA14 assays. 30 μl of the test bacterium were spotted onto the center of the plate and 80 μl of *E. coli* OP50 only were additionally placed in the shape of a ring (Additional file 1: Figure S1). This ring of OP50 protects escaping worms from starvation, minimizing their return to the lawn in the center. The test bacterium consisted of either *B. thuringiensis* diluted with OP50 at 1:250 from a stock with a concentration of 1.5 × 10^10^ particles/ml, or PA14 diluted with OP50 in a 4:1 ratio. 10 hermaphroditic fourth instar larvae (L4) were picked onto the test lawn. Experiments were performed at 20 °C.

Leaving behavior was recorded by counting the number of worms on the lawn at different time points of exposure and calculated according to the following formula:

$$ \mathrm{Leaving}\kern0.5em \mathrm{index}=\frac{10-\mathrm{number}\ \mathrm{of}\ \mathrm{worms}\ \mathrm{on}\ \mathrm{the}\ \mathrm{lawn}}{10} $$

The screens of RILs (200 lines) and ILs (90 lines) were done using a randomized block design on 17 dates, always including the parental strains of these lines, N2 and CB4856, as internal controls. Each *C. elegans* strain-bacteria-time point combination was assayed in three replicates, resulting in a total of 35040 individual data points. The screens of *npr-1* mutants included 12 replicates of each treatment.

We would like to note that the leaving assay for PA14 was performed at 20 °C and thus at a different temperature than the standard PA14 survival assays at 25 °C (see below and [19]). The reason is that the 25 °C temperature led to increased bacterial growth on the assay plate, which caused enhanced dispersal of bacterial colonies through the crawling worms, thus compromising reliable scoring of the leaving behavior. Such a bias was not observed at 20 °C. As our results did confirm previously published data on *C. elegans* avoidance behavior towards PA14 [19], our assay conditions allowed us to characterize a robust behavioral response against this pathogen.

### Survival assays

For survival analysis with *B. thuringiensis*, 6 cm peptone free NGM plates were inoculated with 100 μl of a mixture of *B. thuringiensis* with *E. coli* OP50. Mixtures were prepared in seven dilutions: 1:2, 1:5, 1:10, 1:30, 1:50, 1:100, and 1:250 (equivalent to the relative concentration given in the main text). Plates were left to dry overnight (9–15 h) at 20 °C. 30 L4 hermaphrodites were picked onto each assay plates. After 24 h, survival was recorded by counting alive, dead and missing worms. Each treatment group was replicated 8 times across 8 runs (one replicate per run).

Analysis of *P. aeruginosa* effects was based on 3 cm peptone-rich NGM plates, which were inoculated with 5 μl of an overnight culture at 37 °C of either PA14 or OP50. Seeded plates were incubated overnight at 37 °C and then at 25 °C. 30 L4 hermaphrodites were picked onto each assay plate and stored at 25 °C in the dark. Alive and dead worms were scored every 24 h and surviving worms were transferred to new assay plates every 48 h. Each treatment group was replicated 10 times across two runs.

### Statistical analysis of phenotypic data

We used the non-parametric Kruskall-Wallis test to assess differences in leaving behavior between the *C. elegans* strains, and a Bonferroni based adjustment to correct for multiple testing. We used Kaplan Meier analysis applying the Log Rank test to assess differences in *C. elegans*’ survival on PA14. A Bonferroni based adjustment was used to correct for multiple testing. We used GLM ordinal logistic regression analysis to assess differences in survival between the *C. elegans* strains across the concentration range of *B. thuringiensis*, using *C. elegans* strains, BT concentration and the interaction between the two as factors. A Bonferroni based adjustment was used to correct for multiple testing. All escape and survival assays data were analyzed using the program JMP version 9.0 (SAS Institute Inc.), while graphic illustrations were produced with the program SIGMAPLOT version 12.0 (Systat Software Inc.).

### Quantitative trait locus (QTL) analysis

The QTL analysis was performed on the average of three replicates per genotype/line of the calculated proportion “leave” (see assay method) of 200 Recombinant Inbred Lines (RILs) [41, 55–58, 73–76]. QTLs were calculated by single marker mapping using a linear model (trait ~ marker + error) for each marker using a custom written script in the statistical programming language “R” [77]. Significance levels were estimated from 1000 permutations of the data. The analysis calculated the significance of the linkage between the genetic marker and the trait one by one for each bacterium and exposure time point separately. We furthermore evaluated epistatic interactions between each two markers across the genome for each bacterium separately using the following model: phenotype ~ time + marker1 * marker2. BIN mapping in the set of 90 ILs was done as described in [42, 56].

### RNA isolation and sequencing

N2 and *npr-1(ur89)* worms were exposed to either PA14, BT B-18247 or OP50 for either 12 or 24 h of exposure. The experiment had 3 replicates of each treatment combination (a total of 36 samples across treatment combinations and replicates). Exposure experiments were performed on large Agar plates (15 cm diameter), which were fully covered with a bacterial lawn, thus minimizing escape responses and resulting in comparable occupancy rates across the treatment combinations. At both time points (12 & 24), worms were washed off the exposure plates with PBS containing 0.3 % Tween20® and resuspended in TRIzol® (Life Technologies) reagent. Prior to RNA extraction, worm suspensions were treated five times with a freeze-thaw cycle using liquid nitrogen and a thermo block at 45 °C. RNA extraction was performed using a NucleoSpin® mRNA extraction kit (Macherey-Nagel). RNA samples were treated with DNAse, and then stored at –80 °C. RNA libraries were prepared for sequencing using standard Illumina protocols. Libraries were sequenced on an Illumina HiSeq™ 2000 sequencing machine with a paired-end strategy and read length of 100 nucleotides.

### Statistical analysis of transcriptomic data

RNAseq reads were mapped to the *C. elegans* genome from Wormbase version WS235 (www.wormbase.org) by Tophat2 [78] using option *--b2-very-sensitive,* other default settings and without a transcriptome reference. Tophat2 aligns RNAseq reads to a genome based on the ultra-fast short read mapping program Bowtie [79]. Estimation of transcript abundance and significantly differentially expressed genes were identified by Cuffdiff [80] using the quartile normalization method [81]. Cuffdiff is a program from the Cufflinks package and aims to find significant changes in transcript expression in consideration of possible formation of isoforms for a particular gene. The raw data is available from the GEO database [82, 83] under the GSE number GSE60063.

For clustering and visualization, transcripts with a significant change between different conditions (adjusted *p*-value < 0.01 by the false discovery rate, FDR) were treated as signature for each comparison. Due to the biological variation of the replicates, the *p*-value, instead of fold-change, of those genes were firstly log10-transformed and ordered according to increasing or decreasing expression and then taken as input for k-means cluster analysis using cluster 3.0 [84] with a k of 8. A heatmap was generated by TreeView version 1.1.4r3 [85]. Principal component analysis (PCA) was carried out on log-transformed gene expression profiles using a probabilistic PCA algorithm [86] from R package pcaMethods [87], which links PCA to the probability density of patterns. Dimensionality of the samples was reduced from 57165 (total isoforms) to three dimensions (PCs). Motif analysis was carried out on the promoter regions, -600 bp and 250 bp relative to transcription start sites (TSS), of genes in each group. De novo motif discovery was performed by AMD [54].

### GO and EASE analysis

Gene ontology (GO) and a gene set enrichment analysis was carried out on each group of genes from the K means cluster analysis. GO analysis was performed using DAVID [52, 53] with a cut-off of *p*-value < 0.05, adjusted by FDR. For the gene set analysis, we used EASE [50], a free, stand-alone software package from DAVID bioinformatics resources (http://david.abcc.ncifcrf.gov/). As recently described [51, 64], we constructed a *C. elegans*-specific gene set database, WormExp [51], from published data and also using the previously established data sets collected by Ilka Engelmann et al., [88]. Based on this data set, we performed the EASE analysis and selected the results with a Bonferroni adjusted *p*-value < 0.05.

### eQTL enrichment analysis

eQTL enrichment was done using a hypergeometric test in R. The eQTL sets [41, 57–59] were obtained from WormQTL.org [55, 60]. The eQTLs at a specific locus were compared to the genes in a specific expression cluster, as identified from the above described K-means cluster analysis.

### Ethics approval and consent to participate

Not applicable.

### Consent for publication

Not applicable.

### Availability of data and material

The datasets, supporting the conclusions of this article, is available in case of the QTL analysis from WormQTL [55, 60], in case of the phenotypic analysis of N2, CB4856, and the *npr-1* mutants in Additional file 2 of this article, and in case of the transcriptome analysis from the GEO database [82, 83] under the GSE number GSE60063. The *C. elegans* strains are available from the Caenorhabditis Genetic Center (CGC), which is funded by NIH Office of Research Infrastructure Programs (P40 OD010440). All bacterial strains are available from the corresponding author upon request.

## Additional files

Additional file 1: Illustration of the lawn leaving assay. 10 hermaphrodites at the L4 stage were transferred by picking onto 9 cm peptone free NGM plates containing a lawn of the tested bacteria, each mixed with *E. coli* OP50 and surrounded by a ring of 80 μl of OP50. (TIF 144 kb) Additional file 2: Raw data for the analysis of N2, CB4856, and the npr-1 mutants. Sheet 1, Lawn leaving behavior of N2 and CB4856 from Bacillus thuringiensis - Raw data for Fig. 1; sheet 2, Lawn leaving behavior of mutants from Bacillus thuringiensis - Raw data for Fig. 4; sheet 3, Surival of N2 and CB4856 on Bacillus thuringiensis - Raw data for Additional file 9; sheet 4, Surival of mutants on Bacillus thuringiensis - Raw data for Fig. 5; sheet 5, Lawn leaving behavior of N2 and CB4856 from Pseudomonas aeruginosa PA14 - Raw data for Additional file 10; sheet 6, Lawn leaving behavior of mutants from Pseudomonas aeruginosa PA14 - Raw data for Fig. 6; sheet 7, Surival of mutants on Pseudomonas PA14 - Raw data for Fig. 5. (XLS 482 kb) Additional file 3: Table on the statistical results for the comparison between N2 and CB4856 leaving behavior towards *B. thuringiensis* and *E. coli.* (PDF 83 kb) Additional file 4: Table on the statistical results for the pairwise comparisons of the leaving response on the *E.coli* OP50 control versus the *B. thuringiensi*s strains. (PDF 87 kb) Additional file 5: Figure on the leaving phenotypes of the introgression lines (ILs) plotted against the introgression position along the chromosomes. (A) Results for *E. coli* strain OP50; (B) non-nematocidal *B. thuringiensis* strain DSM350; (C) nematocidal *B. thuringiensis* B-18247; and (D) highly nematocidal *B. thuringiensis* B-18679. Green and red lines show the results after either 14 h or 24 h exposure, respectively. Position of markers is given along the X axis. Light gray vertical lines indicate boundaries of the chromosomes. (TIF 1279 kb) Additional file 6: Table on the statistical results for the comparison of the N2 and CB4856 leaving behavior with that of the mutant strains towards *B. thuringiensis* and *E. coli.* (PDF 96 kb) Additional file 7: Table on the statistical results for the separate comparison between *C. elegans* N2 and CB4856 survival on nematocidal *B. thuringiensis.* (PDF 74 kb) Additional file 8: Table on the statistical results for the pairwise comparison of survival of the *C. elegans* strains on nematocidal *B. thuringiensis.* (PDF 78 kb) Additional file 9: Figure on the separate analysis of N2 and CB4856 survival in the presence of nematocidal *B. thuringiensis*. (A) Survival on *B. thuringiensis* strain B-18247; and (B) B-18679. Survival on the Y axis is plotted against BT concentration on the X axis. Error bars represent standard error of the means. The statistical results are given in Additional file 6. (TIF 106 kb) Additional file 10: Figure on the separate analysis of lawn leaving behavior of N2 and CB4856 towards *E. coli* and *P. aeruginosa*. (A) Results for avoidance of *E. coli* strain OP50; and (B) *P. aeruginosa* strain PA14. The asterisk (*) points to a significant difference to N2. The dotted reference line indicates the 0.5 avoidance response. Statistical results are shown in Additional file 10. (TIF 97 kb) Additional file 11: Table on the statistical results for the comparison between N2 and CB4856 leaving behavior towards *E. coli* and *P. aeruginosa*. (PDF 76 kb) Additional file 12: Table on the statistical results for the comparison of the N2 and CB4856 leaving behavior with that of the mutant strains towards *P. aeruginosa* and *E. coli*. (PDF 90 kb) Additional file 13: Table on the statistical results for the comparison of *C. elegans* survival rate on *P. aeruginosa* PA14 versus the control *E. coli* OP50. (PDF 74 kb) Additional file 14: Table on the statistical results for the pairwise comparisons of *C. elegans* survival rates on *P. aeruginosa* PA14. (PDF 72 kb) Additional file 15: Table with the list of differentially expressed genes after exposure of the *C. elegans* N2 wildtype or *npr-1* mutant to pathogenic *B. thuringiensis* B-18247, pathogenic *P. aeruginosa* PA14, or non-pathogenic *E. coli*. (XLS 2096 kb) Additional file 16: Results of the eQTL enrichment analysis of the K means clusters of differentially expressed genes. (XLS 160 kb) Additional file 17: Results of the GO term enrichment analysis (first sheet), the motif analysis (second sheet), and the *C. elegans*-specific EASE enrichment analysis (third sheet) of the K means clusters of differentially expressed genes. (XLS 316 kb)

## Abbreviations

AMD: automated motif discovery
DAVID: database for annotation, visualization and integrated discovery
EASE: expression analysis systematic explorer
eQTL: expression quantitative trait locus
FDR: false discovery rate
GEO: gene expression omnibus
GLM: generalized linear model
GO: gene ontology
PCA: principal component analysis
QTL: quantitative trait locus

## References

[1] Schulenburg H, Boehnisch C, Michiels NK (2007). How do invertebrates generate a highly specific innate immune response?. Mol Immunol.

[2] Irazoqui JE, Urbach JM, Ausubel FM (2010). Evolution of host innate defence: insights from *Caenorhabditis elegans* and primitive invertebrates. Nat Rev Immunol.

[3] Buchon N, Silverman N, Cherry S (2014). Immunity in *Drosophila melanogaster -* from microbial recognition to whole-organism physiology. Nat Rev Immunol.

[4] Meisel JD, Kim DH (2014). Behavioral avoidance of pathogenic bacteria by *Caenorhabditis elegans*. Trends Immunol.

[5] Ewbank JJ, Pujol N (2016). Local and long-range activation of innate immunity by infection and damage in *C. elegans*. Curr Opin Immunol.

[6] Luhachack LG, Visvikis O, Wollenberg AC, Lacy-Hulbert A, Stuart LM, Irazoqui JE (2012). EGL-9 controls *C. elegans* host defense specificity through prolyl hydroxylation-dependent and-independent HIF-1 pathways. PLoS Pathog.

[7] Shao Z, Zhang Y, Ye Q, Saldanha JN, Powell-Coffman JA (2010). *C. elegans* SWAN-1 Binds to EGL-9 and regulates HIF-1-mediated resistance to the bacterial pathogen Pseudomonas aeruginosa PAO1. PLoS Pathog.

[8] Bellier A, Chen C-S, Kao C-Y, Cinar HN, Aroian RV (2009). Hypoxia and the hypoxic response pathway protect against pore-forming toxins in *C. elegans*. PLoS Pathog.

[9] Tenor JL, Aballay A (2008). A conserved Toll-like receptor is required for *Caenorhabditis elegans* innate immunity. EMBO Rep.

[10] Pujol N, Link EM, Liu LX, Kurz CL, Alloing G, Tan M-W, Ray KP, Solari R, Johnson CD, Ewbank JJ. A reverse genetic analysis of components of the Toll signaling pathway in *Caenorhabditis elegans*. Curr Biol. 2001;11(11):809–21. 10.1016/s0960-9822(01)00241-x 11516642

[11] Schulenburg H, Ewbank JJ (2007). The genetics of pathogen avoidance in *Caenorhabditis elegans*. Mol Microbiol.

[12] Petersen C, Dirksen P, Schulenburg H (2015). Why we need more ecology for genetic models such as *C. elegans*. Trends Genet.

[13] Felix MA, Braendle C (2010). The natural history of *Caenorhabditis elegans*. Curr Biol.

[14] Felix M-A, Duveau F (2012). Population dynamics and habitat sharing of natural populations of Caenorhabditis elegans and *C. briggsae*. BMC Biol.

[15] Petersen C, Dirksen P, Prahl S, Strathmann EA, Schulenburg H (2014). The prevalence of *Caenorhabditis elegans* across 1.5 years in selected North German locations: the importance of substrate type, abiotic parameters, and Caenorhabditis competitors. BMC Ecol.

[16] Pradel E, Zhang Y, Pujol N, Matsuyama T, Bargmann CI, Ewbank JJ (2007). Detection and avoidance of a natural product from the pathogenic bacterium *Serratia marcescens* by *Caenorhabditis elegans*. Proc Natl Acad Sci U S A.

[17] Reddy KC, Andersen EC, Kruglyak L, Kim DH (2009). A polymorphism in *npr-1* is a behavioral determinant of pathogen susceptibility in *C. elegans*. Science.

[18] Styer KL, Singh V, Macosko E, Steele SE, Bargmann CI, Aballay A (2008). Innate immunity in *Caenorhabditis elegans* is regulated by neurons expressing NPR-1/GPCR. Science.

[19] Chang HC, Paek J, Kim DH (2012). Natural polymorphisms in *C. elegans* HECW-1 E3 ligase affect pathogen avoidance behaviour. Nature.

[20] McMullan R, Anderson A, Nurrish S (2012). Behavioral and immune responses to infection require Gαq- RhoA Signaling in *C. elegans*. PLoS Pathog.

[21] Hasshoff M, Boehnisch C, Tonn D, Hasert B, Schulenburg H (2007). The role of *Caenorhabditis elegans* insulin-like signaling in the behavioral avoidance of pathogenic *Bacillus thuringiensis*. FASEB J.

[22] Zhang Y, Lu H, Bargmann CI (2005). Pathogenic bacteria induce aversive olfactory learning in *Caenorhabditis elegans*. Nature.

[23] Schulenburg H, Muller S (2004). Natural variation in the response of *Caenorhabditis elegans* toward *Bacillus thruingiensis*. Parasitology.

[24] Volkers RJM, Snoek LB, van Hellenberg Hubar CJ, Coopman R, Chen W, Yang W, Sterken MG, Schulenburg H, Braeckman BP, Kammenga JE. Gene-environment and protein-degradation signatures characterize genomic and phenotypic diversity in wild *Caenorhabditis elegans* populations. BMC Biol. 2013;11(1):93. 10.1186/1741-7007-11-93 PMC3846632 23957880

[25] Sicard M, Hering S, Schulte R, Gaudriault S, Schulenburg H (2007). The effect of *Photorhabdus luminescens* (Enterobacteriaceae) on the survival, development, reproduction and behaviour of *Caenorhabditis elegans* (Nematoda: Rhabditidae). Environ Microbiol.

[26] Glater EE, Rockman MV, Bargmann CI (2014). Multigenic natural variation underlies *Caenorhabditis elegans* olfactory preference for the bacterial pathogen *Serratia marcescens*. G3.

[27] Andersen EC, Gerke JP, Shapiro JA, Crissman JR, Ghosh R, Bloom JS, Felix M-A, Kruglyak L. Chromosome-scale selective sweeps shape *Caenorhabditis elegans* genomic diversity. Nat Genet. 2012;44(3):285–90. 10.1038/ng.1050 PMC3365839 22286215

[28] de Bono M, Bargmann CI (1998). Natural variation in a neuropeptide Y receptor homolog modifies social behavior and food response in *C. elegans*. Cell.

[29] Rogers C, Persson A, Cheung B, de Bono M (2006). Behavioral motifs and neural pathways coordinating O2 responses and aggregation in *C. elegans*. Curr Biol.

[30] Weber KP, De S, Kozarewa I, Turner DJ, Babu MM, de Bono M (2010). Whole genome sequencing highlights genetic changes associated with laboratory domestication of C. elegans. PLoS One.

[31] Bendesky A, Tsunozaki M, Rockman MV, Kruglyak L, Bargmann CI (2012). Catecholamine receptor polymorphisms affect decision-making in *C. elegans*. Nature.

[32] Borgonie G, Van Driessche R, Leyns F, Arnaut G, De Waele D, Coomans A (1995). Germination of *Bacillus thuringiensis s*pores in bacteriophagous nematodes (Nematoda: Rhabditida). J Invertebr Pathol.

[33] Borgonie G, Claeys M, Leyns F, Arnaut G, De Waele D, Coomans AV (1996). Effect of a nematicidal *Bacillus thuringiensis* strain on free-living nematodes. 3. Characterization of the intoxication process. Fundam Appl Nematol.

[34] Wei JZ, Hale K, Carta L, Platzer E, Wong C, Fang SC, Aroian RV. *Bacillus thuringiensis* crystal proteins that target nematodes. Proc Natl Acad Sci U S A. 2003;100(5):2760–5. 10.1073/pnas.0538072100 PMC151414 12598644

[35] Griffitts JS, Aroian RV (2005). Many roads to resistance: how invertebrates adapt to Bt toxins. Bioessays.

[36] Nielsen-LeRoux C, Gaudriault S, Ramarao N, Lereclus D, Givaudan A (2012). How the insect pathogen bacteria *Bacillus thuringiensis* and Xenorhabdus/Photorhabdus occupy their hosts. Curr Opin Microbiol.

[37] Wang J, Nakad R, Schulenburg H (2012). Activation of the *Caenorhabditis elegans* FOXO family transcription factor DAF-16 by pathogenic *Bacillus thuringiensis*. Dev Comp Immunol.

[38] Schulte RD, Makus C, Hasert B, Michiels NK, Schulenburg H (2011). Host–parasite local adaptation after experimental coevolution of *Caenorhabditis elegans* and its microparasite *Bacillus thuringiensis*. Proc Royal Soc B.

[39] Schulte RD, Makus C, Hasert B, Michiels NK, Schulenburg H (2010). Multiple reciprocal adaptations and rapid genetic change upon experimental coevolution of an animal host and its microbial parasite. Proc Natl Acad Sci.

[40] Masri L, Branca A, Sheppard AE, Papkou A, Laehnemann D, Guenther PS, Prahl S, Saebelfeld M, Hollensteiner J, Liesegang H. Host-pathogen coevolution: the selective advantage of *Bacillus thuringiensis* virulence and its cry toxin genes. PLoS Biol. 2015;13(6):e1002169. 10.1371/journal.pbio.1002169 PMC4456383 26042786

[41] Li Y, Ãlvarez OA, Gutteling EW, Tijsterman M, Fu J, Riksen JAG, Hazendonk E, Prins P, Plasterk RHA, Jansen RC, et al. Mapping determinants of gene expression plasticity by genetical genomics in *C. elegans*. PLoS Genet. 2006;2(12):e222. 10.1371/journal.pgen.0020222 PMC1756913 17196041

[42] Doroszuk A, Snoek LB, Fradin E, Riksen J, Kammenga J (2009). A genome-wide library of CB4856/N2 introgression lines of *Caenorhabditis elegans*. Nucleic Acids Res.

[43] Balla KM, Troemel ER (2015). *Caenorhabditis elegans* as a model for intracellular pathogen infection. Cell Microbiol.

[44] Wilfert L, Gadau J, Schmid‐Hempel P (2007). The genetic architecture of immune defense and reproduction in male *Bombus terrestris* bumblebees. Evolution.

[45] Luijckx P, Fienberg H, Duneau D, Ebert D (2013). A matching-allele model explains host resistance to parasites. Curr Biol.

[46] Schulenburg H, Kurtz J, Moret Y, Siva-Jothy MT (2009). Introduction. Ecological immunology. Philo Transac Royal Soc B.

[47] Ferrandon D, Imler J-L, Hetru C, Hoffmann JA (2007). The Drosophila systemic immune response: sensing and signalling during bacterial and fungal infections. Nat Rev Immunol.

[48] Cheung BHH, Cohen M, Rogers C, Albayram O, de Bono M (2005). Experience-dependent modulation of *C. elegans* behavior by ambient oxygen. Curr Biol.

[49] Chang AJ, Chronis N, Karow DS, Marletta MA, Bargmann CI (2006). A distributed chemosensory circuit for oxygen preference in *C. elegans*. PLoS Biol.

[50] Hosack DA, Dennis G, Sherman BT, Lane HC, Lempicki RA (2003). Identifying biological themes within lists of genes with EASE. Genome Biol.

[51] Yang W, Dierking K, Schulenburg H. WormExp: a web-based application for a *Caenorhabditis elegans*-specific gene expression enrichment analysis. Bioinformatics. 2015; [Epub ahead of print]. 10.1093/bioinformatics/btv667 26559506

[52] Huang DW, Sherman BT, Lempicki RA (2008). Systematic and integrative analysis of large gene lists using DAVID bioinformatics resources. Nat Protoc.

[53] Huang DW, Sherman BT, Lempicki RA (2009). Bioinformatics enrichment tools: paths toward the comprehensive functional analysis of large gene lists. Nucleic Acids Res.

[54] Shi J, Yang W, Chen M, Du Y, Zhang J, Wang K (2011). AMD, an automated motif discovery tool using stepwise refinement of gapped consensuses. PLoS One.

[55] Snoek LB, Van der Velde KJ, Arends D, Li Y, Beyer A, Elvin M, Fisher J, Hajnal A, Hengartner MO, Poulin GB, et al. Worm QTL—public archive and analysis web portal for natural variation data in Caenorhabditis spp. Nucleic Acids Res. 2013;41(D1):D738–43. 10.1093/nar/gks1124 PMC3531126 23180786

[56] Snoek LB, Orbidans HE, Stastna JJ, Aartse A, Rodriguez M, Riksen JAG, Kammenga JE, Harvey SC. Widespread genomic incompatibilities in *Caenorhabditis elegans*. G3. 2014;4(10):1813–23. 10.1534/g3.114.013151 PMC4199689 25128438

[57] Li Y, Breitling R, Snoek LB, Velde KJ, Swertz MA, Riksen JAG, Jansen RC, Kammenga JE. Global genetic robustness of the alternative splicing machinery in *Caenorhabditis elegans*. Genetics. 2010;186:405–10. 10.1534/genetics.110.119677 PMC2940304 20610403

[58] Viñuela A, Snoek LB, Riksen JAG, Kammenga JE (2010). Genome-wide gene expression regulation as a function of genotype and age in *C. elegans*. Genome Res.

[59] Rockman MV, Skrovanek SS, Kruglyak L (2010). Selection at linked sites shapes heritable phenotypic variation in *C. elegans*. Science.

[60] Snoek LB, Joeri van der Velde K, Li Y, Jansen RC, Swertz MA, Kammenga JE. Worm variation made accessible: Take your shopping cart to store, link, and investigate! In: Worm. Abingdon, UK: Taylor & Francis; 2014: e28357. 10.4161/worm.28357 PMC4024057 24843834

[61] Miltsch SM, Seeberger PH, Lepenies B (2014). The C-type lectin-like domain containing proteins CLEC-39 and CLEC-49 are crucial for *Caenorhabditis elegans* immunity against *Serratia marcescens* infection. Dev Compar Immunol.

[62] Irazoqui JE, Troemel ER, Feinbaum RL, Luhachack LG, Cezairliyan BO, Ausubel FM (2010). Distinct pathogenesis and host responses during infection of *C. elegans* by *P. aeruginosa* and *S. aureus*. PLoS Pathog.

[63] O’Rourke D, Baban D, Demidova M, Mott R, Hodgkin J (2006). Genomic clusters, putative pathogen recognition molecules, and antimicrobial genes are induced by infection of *C. elegans* with *M. nematophilum*. Genome Res.

[64] Yang W, Dierking K, Esser D, Tholey A, Leippe M, Rosenstiel P, Schulenburg H. Overlapping and unique signatures in the proteomic and transcriptomic responses of the nematode *Caenorhabditis elegans* toward pathogenic *Bacillus thuringiensis*. Dev Compar Immunol. 2015;51(1):1–9. 10.1016/j.dci.2015.02.010 25720978

[65] Kim DH, Feinbaum R, Alloing G, Emerson FE, Garsin DA, Inoue H, Tanaka-Hino M, Hisamoto N, Matsumoto K, Tan M-W, et al. A conserved p38 MAP kinase pathway in *Caenorhabditis elegans* innate immunity. Science. 2002;297(5581):623–6. 10.1126/science.1073759 12142542

[66] Shapira M, Hamlin BJ, Rong J, Chen K, Ronen M, Tan M-W (2006). A conserved role for a GATA transcription factor in regulating epithelial innate immune responses. Proc Natl Acad Sci.

[67] Kao C-Y, Los FCO, Huffman DL, Wachi S, Kloft N, Husmann M, Karabrahimi V, Schwartz J-L, Bellier A, Ha C, et al. Global functional analyses of cellular responses to pore-forming toxins. PLoS Pathog. 2011;7(3):e1001314. 10.1371/journal.ppat.1001314 PMC3048360 21408619

[68] Feng N, Zhi D, Zhang L, Tian J, Ren H, Li C, Zhu H, Li H. Molecular mechanisms of resistance to human pathogenic bacteria in *Caenorhabditis elegans* by MEV-1 mediated oxidative stress. Biochem Biophys Res Commun. 2015;459(3):481–7. 10.1016/j.bbrc.2015.02.132 25747713

[69] Schmid-Hempel P (2008). Parasite immune evasion: a momentous molecular war. Trends Ecol Evol.

[70] Niu Q, Huang X, Zhang L, Xu J, Yang D, Wei K, Niu X, An Z, Bennett JW, Zou C. A Trojan horse mechanism of bacterial pathogenesis against nematodes. Proc Natl Acad Sci. 2010;107(38):16631–6. 10.1073/pnas.1007276107 PMC2944701 20733068

[71] Stiernagle T (2006). Maintenance of *C. elegans*. WormBook. The *C. elegans* research community. WormBook.

[72] Leyns F, Borgonie G, Arnaut G, De Waele D (1995). Nematicidal activity of *Bacillus thuringiensis* isolates. Fundam Appl Nematol.

[73] Gutteling EW, Doroszuk A, Riksen JAG, Prokop Z, Reszka J, Kammenga JE (2007). Environmental influence on the genetic correlations between life-history traits in *Caenorhabditis elegans*. Heredity.

[74] Viñuela A, Snoek LB, Riksen JAG, Kammenga JE (2012). Aging uncouples heritability and expression-QTL in *Caenorhabditis elegans*. G3.

[75] Elvin M, Snoek L, Frejno M, Klemstein U, Kammenga J, Poulin G (2011). A fitness assay for comparing RNAi effects across multiple *C. elegans* genotypes. BMC Genomics.

[76] Rodriguez M, Snoek LB, Riksen JAG, Bevers RP, Kammenga JE (2012). Genetic variation for stress-response hormesis in *C. elegans* lifespan. Exp Gerontol.

[77] R: A Language and Environment for Statistical Computing, R Core Team, R Foundation for Statistical Computing, Vienna, Austria, 2015, http://www.R-project.org/. 2015.

[78] Kim D, Pertea G, Trapnell C, Pimentel H, Kelley R, Salzberg SL (2013). TopHat2: accurate alignment of transcriptomes in the presence of insertions, deletions and gene fusions. Genome Biol.

[79] Langmead B, Trapnell C, Pop M, Salzberg SL (2009). Ultrafast and memory-efficient alignment of short DNA sequences to the human genome. Genome Biol.

[80] Trapnell C, Hendrickson DG, Sauvageau M, Goff L, Rinn JL, Pachter L (2013). Differential analysis of gene regulation at transcript resolution with RNA-seq. Nat Biotechnol.

[81] Robinson MD, Oshlack A (2010). A scaling normalization method for differential expression analysis of RNA-seq data. Genome Biol.

[82] Edgar R, Domrachev M, Lash AE (2002). Gene expression omnibus: NCBI gene expression and hybridization array data repository. Nucleic Acids Res.

[83] Barrett T, Wilhite SE, Ledoux P, Evangelista C, Kim IF, Tomashevsky M, Marshall KA, Phillippy KH, Sherman PM, Holko M. NCBI GEO: archive for functional genomics data sets update. Nucleic Acids Res. 2013;41(D1):D991–5. 10.1093/nar/gks1193 PMC3531084 23193258

[84] de Hoon MJ, Imoto S, Nolan J, Miyano S (2004). Open source clustering software. Bioinformatics.

[85] Saldanha AJ (2004). Java Treeview--extensible visualization of microarray data. Bioinformatics.

[86] Tipping ME, Bishop CM (1999). Probabilistic principal component analysis. J Royal Stat Soc Ser B.

[87] Stacklies W, Redestig H, Scholz M, Walther D, Selbig J (2007). pcaMethods--a bioconductor package providing PCA methods for incomplete data. Bioinformatics.

[88] Engelmann I, Griffon A, Tichit L, Montanana-Sanchis F, Wang G, Reinke V, Waterston RH, Hillier LW, Ewbank JJ. A comprehensive analysis of gene expression changes provoked by bacterial and fungal infection in C. elegans. PLoS One. 2011;6(5):e19055. 10.1371/journal.pone.0019055 PMC3094335 21602919

[89] Huffman DL, Abrami L, Sasik R, Corbeil J, van der Goot FG, Aroian RV (2004). Mitogen-activated protein kinase pathways defend against bacterial pore-forming toxins. Proc Natl Acad Sci U S A.

[90] Boehnisch C, Wong D, Habig M, Isermann K, Michiels NK, Roeder T, May RC, Schulenburg H. Protist-type lysozymes of the nematode *Caenorhabditis elegans* contribute to resistance against pathogenic *Bacillus thuringiensis*. PLoS One. 2011;6(9):e24619. 10.1371/journal.pone.0024619 PMC3169628 21931778

[91] Troemel ER, Chu SW, Reinke V, Lee SS, Ausubel FM, Kim DH (2006). p38 MAPK regulates expression of immune response genes and contributes to longevity in *C. elegans*. PLoS Genet.

[92] Park SK, Tedesco PM, Johnson TE (2009). Oxidative stress and longevity in *Caenorhabditis elegans* as mediated by SKN-1. Aging Cell.

[93] Rohlfing A-K, Miteva Y, Hannenhalli S, Lamitina T (2010). Genetic and physiological activation of osmosensitive gene expression mimics transcriptional signatures of pathogen infection in *C. elegans*. PLoS One.

[94] Cui Y, McBride SJ, Boyd WA, Alper S, Freedman JH (2007). Toxicogenomic analysis of *Caenorhabditis elegans* reveals novel genes and pathways involved in the resistance to cadmium toxicity. Genome Biol.

[95] Murphy CT, McCarroll SA, Bargmann CI, Fraser A, Kamath RS, Ahringer J, Li H, Kenyon C. Genes that act downstream of DAF-16 to influence the lifespan of *Caenorhabditis elegans*. Nature. 2003;424(6946):277–83. 10.1038/nature01789 12845331

[96] McElwee JJ, Schuster E, Blanc E, Thomas JH, Gems D (2004). Shared transcriptional signature in *Caenorhabditis elegans* Dauer larvae and long-lived *daf-2* mutants implicates detoxification system in longevity assurance. J Biol Chem.

[97] Halaschek-Wiener J, Khattra JS, McKay S, Pouzyrev A, Stott JM, Yang GS, Holt RA, Jones SJM, Marra MA, Brooks-Wilson AR. Analysis of long-lived *C. elegans daf-2* mutants using serial analysis of gene expression. Genome Res. 2005;15(5):603–15. 10.1101/gr.3274805 PMC1088289 15837805

[98] Kim SK, Lund J, Kiraly M, Duke K, Jiang M, Stuart JM, Eizinger A, Wylie BN, Davidson GS. A gene expression map for *Caenorhabditis elegans*. Science. 2001;293(5537):2087–92. 10.1126/science.1061603 11557892

[99] Menzel R, Bogaert T, Achazi R (2001). A systematic gene expression screen of *Caenorhabditis elegans* cytochrome P450 genes reveals CYP35 as strongly xenobiotic inducible. Arch Biochem Biophys.

[100] Reichert K, Menzel R (2005). Expression profiling of five different xenobiotics using a *Caenorhabditis elegans* whole genome microarray. Chemosphere.

[101] Grove CA, De Masi F, Barrasa MI, Newburger DE, Alkema MJ, Bulyk ML, Walhout AJM. A multiparameter network reveals extensive divergence between *C. elegans* bHLH transcription factors. Cell. 2009;138(2):314–27. 10.1016/j.cell.2009.04.058 PMC2774807 19632181

[102] McGhee JD, Sleumer MC, Bilenky M, Wong K, McKay SJ, Goszczynski B, Tian H, Krich ND, Khattra J, Holt RA. The ELT-2 GATA-factor and the global regulation of transcription in the *C. elegans* intestine. Dev Biol. 2007;302(2):627–45. 10.1016/j.ydbio.2006.10.024 17113066

[103] Niu W, Lu ZJ, Zhong M, Sarov M, Murray JI, Brdlik CM, Janette J, Chen C, Alves P, Preston E. Diverse transcription factor binding features revealed by genome-wide ChIP-seq in *C. elegans*. Genome Res. 2011;21(2):245–54. 10.1101/gr.114587.110 PMC3032928 21177963

