# Supplementary figures and images for: Contrasting invertebrate immune defense behaviors caused by a single gene, the Caenorhabditis elegans neuropeptide receptor gene npr-1
Source: BMC Genomics. 2016 Apr 11;17:280. doi: 10.1186/s12864-016-2603-8 (PMC4827197; doi:10.1186/s12864-016-2603-8)

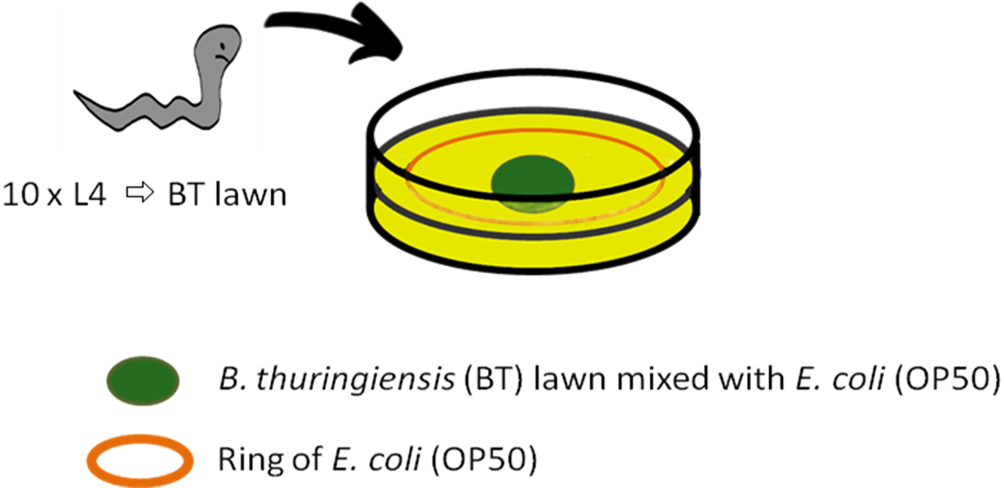

Supplement: Additional file 1: — Illustration of the lawn leaving assay. 10 hermaphrodites at the L4 stage were transferred by picking onto 9 cm peptone free NGM plates containing a lawn of the tested bacteria, each mixed with E. coli OP50 and surrounded by a ring of 80 μl of OP50. (TIF 144 kb) [file 12864_2016_2603_MOESM1_ESM.tif]

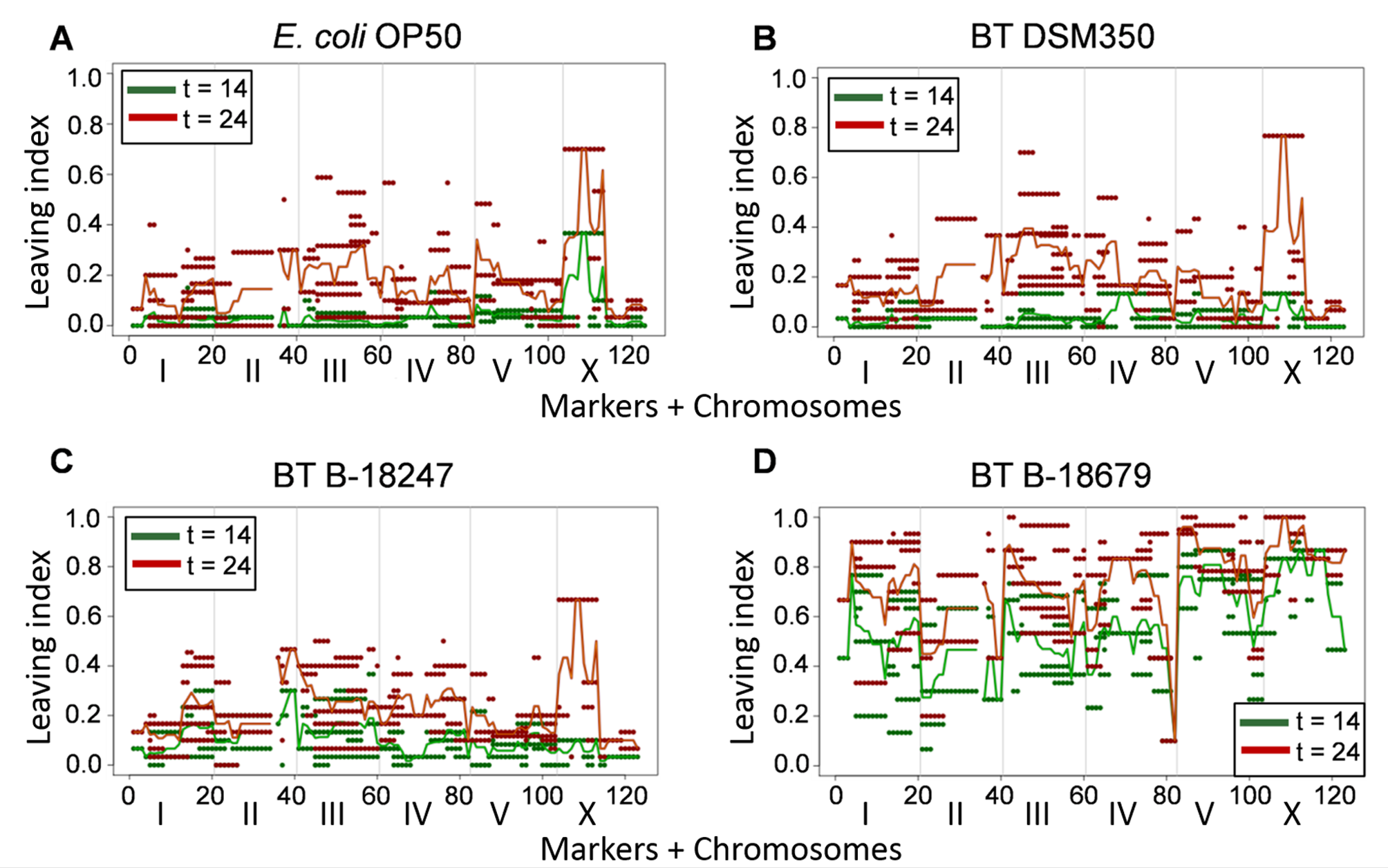

Supplement: Additional file 5: — Figure on the leaving phenotypes of the introgression lines (ILs) plotted against the introgression position along the chromosomes. (A) Results for E. coli strain OP50; (B) non-nematocidal B. thuringiensis strain DSM350; (C) nematocidal B. thuringiensis B-18247; and (D) highly nematocidal B. thuringiensis B-18679. Green and red lines show the results after either 14 h or 24 h exposure, respectively. Position of markers is given along the X axis. Light gray vertical lines indicate boundaries of the chromosomes. (TIF 1279 kb) [file 12864_2016_2603_MOESM5_ESM.tif]

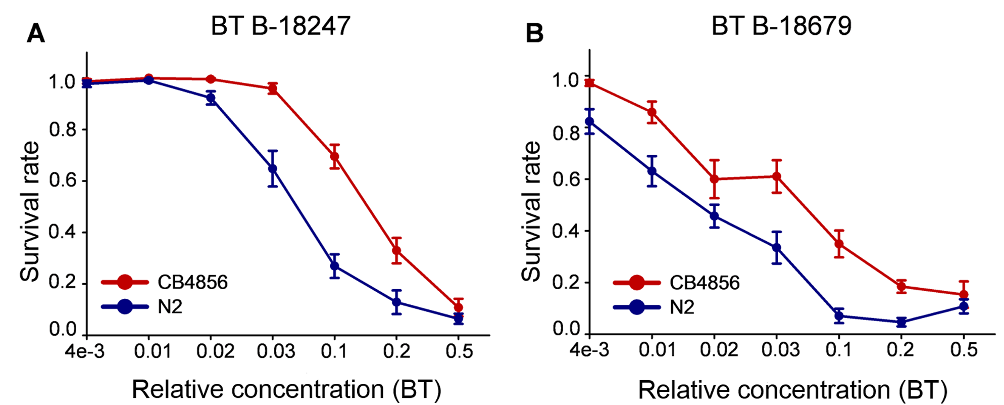

Supplement: Additional file 9: — Figure on the separate analysis of N2 and CB4856 survival in the presence of nematocidal B. thuringiensis. (A) Survival on B. thuringiensis strain B-18247; and (B) B-18679. Survival on the Y axis is plotted against BT concentration on the X axis. Error bars represent standard error of the means. The statistical results are given in Additional file 6. (TIF 106 kb) [file 12864_2016_2603_MOESM9_ESM.tif]

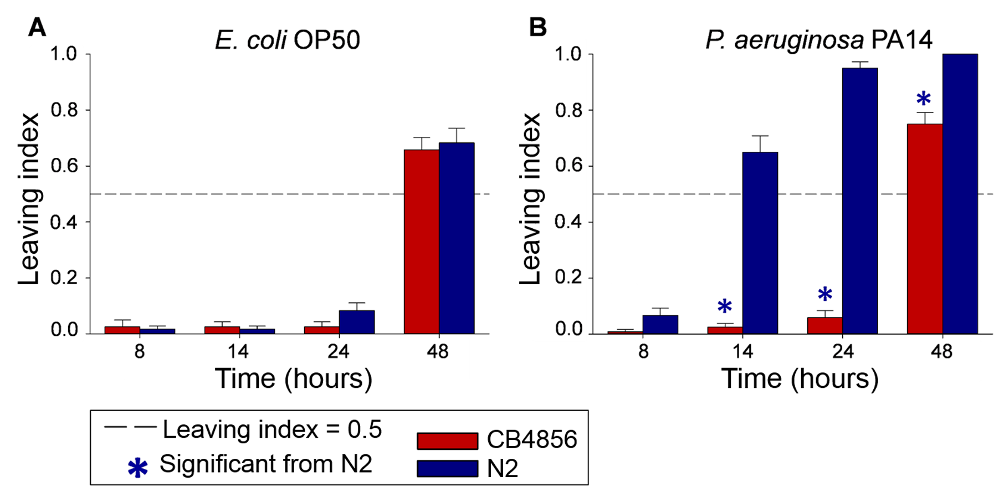

Supplement: Additional file 10: — Figure on the separate analysis of lawn leaving behavior of N2 and CB4856 towards E. coli and P. aeruginosa. (A) Results for avoidance of E. coli strain OP50; and (B) P. aeruginosa strain PA14. The asterisk (*) points to a significant difference to N2. The dotted reference line indicates the 0.5 avoidance response. Statistical results are shown in Additional file 10. (TIF 97 kb) [file 12864_2016_2603_MOESM10_ESM.tif]
