# Supplementary material for: Contrasting invertebrate immune defense behaviors caused by a single gene, the Caenorhabditis elegans neuropeptide receptor gene npr-1
Source: BMC Genomics. 2016 Apr 11;17:280. doi: 10.1186/s12864-016-2603-8 (PMC4827197; doi:10.1186/s12864-016-2603-8)
Supplement: Additional file 3: — Table on the statistical results for the comparison between N2 and CB4856 leaving behavior towards B. thuringiensis and E. coli. (PDF 83 kb) [file 12864_2016_2603_MOESM3_ESM.pdf]

Additional File 2. Table on the statistical results for the comparison between N2 and CB4856 leaving behavior towards *B. thuringiensis* and *E. coli*

**Time point**[1]	**Bacteria**[2]	$\chi 2$	***p***
1 h	B-18247	5.404	0.0201
	B-18679	10.716	**0.0011**
	DSM350	13.931	**0.0002**
	OP50	12.112	**0.0005**
4 h	B-18247	12.398	**0.0004**
	B-18679	5.908	0.0151
	DSM350	15.659	**<0.0001**
	OP50	15.231	**<0.0001**
8 h	B-18247	10.744	**0.001**
	B-18679	16.162	**<0.0001**
	DSM350	19.638	**<0.0001**
	OP50	26.474	**<0.0001**
14 h	B-18247	13.410	**0.0003**
	B-18679	17.698	**<0.0001**
	DSM350	24.956	**<0.0001**
	OP50	24.885	**<0.0001**
24 h	B-18247	17.727	**<0.0001**
	B-18679	9.249	**0.0024**
	DSM350	26.670	**<0.0001**
	OP50	19.416	**<0.0001**

[1] Time point, for each of which the analysis was done separately.

[2] Bacteria, for which the difference between N2 and CB4856 was assessed with the Kruskall-Wallis test, including the nematocidal *B. thuringiensis* B-18247 and B-18679, and the non-nematocidal *B. thuringiensis* DSM350 and *E. coli* OP50. Degrees of freedom (DF) = 1 for all tests. Significant probabilities are given in bold. Significance level was adjusted using Bonferroni correction for multiple pairwise comparisons.
