# Supplementary material for: Contrasting invertebrate immune defense behaviors caused by a single gene, the Caenorhabditis elegans neuropeptide receptor gene npr-1
Source: BMC Genomics. 2016 Apr 11;17:280. doi: 10.1186/s12864-016-2603-8 (PMC4827197; doi:10.1186/s12864-016-2603-8)
Supplement: Additional file 4: — Table on the statistical results for the pairwise comparisons of the leaving response on the E.coli OP50 control versus the B. thuringiensis strains. (PDF 87 kb) [file 12864_2016_2603_MOESM4_ESM.pdf]

Additional File 3. Table on the statistical results for the pairwise comparisons of the leaving response on the *E.coli* OP50 control versus the *B. thuringiensis* strains

		**N2[1]**		**CB4856[1]**	
**Time point[1]**	**Pairwise comparison[2]**	***χ2***	***p***	***χ2***	***p***
1 h	B-18247 *vs.* OP50	0.261	0.6095	0.946	0.3307
	B-18679 *vs.* OP50	1.236	0.2662	0.003	0.9558
	DSM350 *vs.* OP50	0	1	0.028	0.8673
4 h	B-18247 *vs.* OP50	0.617	0.4321	0.370	0.543
	B-18679 *vs.* OP50	15.792	**<0.0001**	1.175	0.2783
	DSM350 *vs.* OP50	0.003	0.9585	0.354	0.5521
8 h	B-18247 *vs.* OP50	0.369	0.5435	2.357	0.1247
	B-18679 *vs.* OP50	26.057	**<0.0001**	21.916	**<0.0001**
	DSM350 *vs.* OP50	0.166	0.6835	1.012	0.3145
14 h	B-18247 *vs.* OP50	7.368	0.0066	1.275	0.2588
	B-18679 *vs.* OP50	29.917	**<0.0001**	26.925	**<0.0001**
	DSM350 *vs.* OP50	0.916	0.3386	3.132	0.0768
24 h	B-18247 *vs.* OP50	3.296	0.0694	0.829	0.3627
	B-18679 *vs.* OP50	23.642	**<0.0001**	10.668	**0.0011**
	DSM350 *vs.* OP50	3.082	0.0792	2.422	0.1197

[1] The analysis was done separately for the time points and the two *C. elegans* strains N2 and CB4856.

[2] Pairwise comparisons of the response to the *E. coli* OP50 control versus three *B. thuringiensis* strains using the Kruskall-Wallis test. B-18247 and B-18679 are nematocidal; DSM350 is not. Degrees of freedom (DF) = 1 for all tests. Significant probabilities are given in bold. Significance level was adjusted using Bonferroni correction for multiple pairwise comparisons.
