# Supplementary material for: Contrasting invertebrate immune defense behaviors caused by a single gene, the Caenorhabditis elegans neuropeptide receptor gene npr-1
Source: BMC Genomics. 2016 Apr 11;17:280. doi: 10.1186/s12864-016-2603-8 (PMC4827197; doi:10.1186/s12864-016-2603-8)
Supplement: Additional file 6: — Table on the statistical results for the comparison of the N2 and CB4856 leaving behavior with that of the mutant strains towards B. thuringiensis and E. coli. (PDF 96 kb) [file 12864_2016_2603_MOESM6_ESM.pdf]

Additional File 5. Table on the statistical results for the comparison of the N2 and CB4856 leaving behavior with that of the mutant strains towards *B. thuringiensis* and *E. coli*

		**14 h**		**24 h**	
**Comparison**[1]	**Bacteria**	***χ2***	***p***	***χ2***	***p***
N2 vs. *npr-1(ur89)*	B-18247	10.036	**0.0015**	11.108	**0.0009**
	B-18679	3.463	0.0628	1.068	0.3013
	DSM350	14.264	**0.0002**	12.707	**0.0004**
	OP50	10.091	**0.0015**	18.410	**<0.0001**
N2 vs. *npr-1(ad609)*	B-18247	0.003	0.9545	0.094	0.7595
	B-18679	2.696	0.1006	0.540	0.4626
	DSM350	0.023	0.8796	0.400	0.527
	OP50	0.898	0.3434	1.528	0.2164
N2 vs. *tyra-3(ok325)*	B-18247	0.370	0.5426	0.030	0.8618
	B-18679	1.741	0.187	0.036	0.8477
	DSM350	4.924	0.0265	1.438	0.203
	OP50	0.698	0.4033	2.615	0.1058
N2 vs. CB4856	B-18247	1.350	0.2453	19.492	**<0.0001**
	B-18679	0.006	0.9382	1.7789	0.4823
	DSM350	0.395	0.531	8.8199	**0.003**
	OP50	9.318	**0.0023**	17.922	**<0.0001**
CB4856 vs. *npr-1(ur89)*	B-18247	4.200	0.0404	0.2998	0.584
	B-18679	3.713	0.054	0.3005	0.5836
	DSM350	9.007	**0.0027**	1.2515	0.2633
	OP50	0.105	0.7451	1.9035	0.1677
CB4856 vs. *npr-1(ad609)*	B-18247	1.730	0.1884	13.036	**0.0003**
	B-18679	2.454	0.1171	0.7438	0.3885
	DSM350	0.206	0.6494	10.665	**0.0011**
	OP50	5.117	0.0237	20.025	**<0.0001**
CB4856 vs. *tyra-3(ok325)*	B-18247	0.442	0.505	19.391	**<0.0001**
	B-18679	1.364	0.2427	1.146	0.2842
	DSM350	6.838	**0.0089**	10.124	**0.0015**
	OP50	10.935	**0.0009**	18.559	**<0.0001**

[1] The analysis was performed separately for the pairwise comparisons, bacteria, and the two time points. The difference between nematode strains was assessed with the Kruskall Wallis test. The bacteria included the nematocidal *B. thuringiensis* B-18247 and B-18679, and the non-nematocidal *B. thuringiensis* DSM350 and *E. coli* OP50. Degrees of freedom (DF) = 1 for all tests. Significant probabilities are given in bold. Significance level was adjusted using Bonferroni correction for multiple pairwise comparisons.
