# Supplementary material for: Contrasting invertebrate immune defense behaviors caused by a single gene, the Caenorhabditis elegans neuropeptide receptor gene npr-1
Source: BMC Genomics. 2016 Apr 11;17:280. doi: 10.1186/s12864-016-2603-8 (PMC4827197; doi:10.1186/s12864-016-2603-8)
Supplement: Additional file 7: — Table on the statistical results for the separate comparison between C. elegans N2 and CB4856 survival on nematocidal B. thuringiensis. (PDF 74 kb) [file 12864_2016_2603_MOESM7_ESM.pdf]

Additional File 6. Table on the statistical results for the separate comparison between *C. elegans* N2 and CB4856 survival on nematocidal *B. thuringiensis*

**Bacteria**[1]	$\chi 2$	***p***
B-18247	4.36	**0.0367**
B-18679	4.6	**0.0319**

[1] The analysis was performed separately for each bacterium, using ordinal logistic regression, including the following factors: *C. elegans* strains (only N2 and CB4856), *B. thuringiensis* concentration, and the interaction between the two. Effect tests were performed for all factors, but the results are only shown for the *C. elegans* strain comparison (DF = 1). The factor *B. thuringiensis* always had a significant influence, while the interaction term was never significant. Significant probabilities are given in bold.
