# Supplementary material for: Contrasting invertebrate immune defense behaviors caused by a single gene, the Caenorhabditis elegans neuropeptide receptor gene npr-1
Source: BMC Genomics. 2016 Apr 11;17:280. doi: 10.1186/s12864-016-2603-8 (PMC4827197; doi:10.1186/s12864-016-2603-8)
Supplement: Additional file 8: — Table on the statistical results for the pairwise comparison of survival of the C. elegans strains on nematocidal B. thuringiensis. (PDF 78 kb) [file 12864_2016_2603_MOESM8_ESM.pdf]

Additional File 7. Table on the statistical results for the pairwise comparison of survival of the *C. elegans* strains on nematocidal *B. thuringiensis*

**Comparison**[1]	**Bacteria**	**$\chi 2$**	***p***
N2 vs. *npr-1(ur89)*	B-18247	6.6	**0.0102**
	B-18679	14.42	**0.0001**
N2 vs. *npr-1(ad609)*	B-18247	0.3	0.5813
	B-18679	1.27	0.2584
N2 vs. CB4856	B-18247	16.8	**<0.0001**
	B-18679	14.5	**0.0001**
CB4856 vs. *npr-1(ur89)*	B-18247	2.81	0.0931
	B-18679	0.013	0.9068
CB4856 vs. *npr-1(ad609)*	B-18247	15.33	**<0.0001**
	B-18679	20.17	**<0.0001**

[1] The analysis was performed separately for each bacterium and nematode pairwise comparison, using ordinal logistic regression, including the following factors: *C. elegans* strains (only the two considered for the pairwise comparison), *B. thuringiensis* concentration, and the interaction between the two. Effect tests were performed for all factors, but the results are only shown for the *C. elegans* strain comparison (DF = 1). The factor *B. thuringiensis* always had a significant influence, while the interaction term was never significant. The significance level was adjusted across pairwise comparisons using the Bonferroni correction. Significant probabilities are given in bold.
