# Supplementary material for: Contrasting invertebrate immune defense behaviors caused by a single gene, the Caenorhabditis elegans neuropeptide receptor gene npr-1
Source: BMC Genomics. 2016 Apr 11;17:280. doi: 10.1186/s12864-016-2603-8 (PMC4827197; doi:10.1186/s12864-016-2603-8)
Supplement: Additional file 11: — Table on the statistical results for the comparison between N2 and CB4856 leaving behavior towards E. coli and P. aeruginosa. (PDF 76 kb) [file 12864_2016_2603_MOESM11_ESM.pdf]

Additional File 10. Table on the statistical results for the comparison between N2 and CB4856 leaving behavior towards *E. coli* and *P. aeruginosa*

Time point[1]	Bacteria[2]	$\chi 2$	*p*
8 h	PA14	3.7681	0.0522
	OP50	0.2525	0.6153
14 h	PA14	18.4	**<0.0001**
	OP50	0.0079	0.929
24 h	PA14	18.5028	**<0.0001**
	OP50	3.0023	0.0831
48 h	PA14	15.1565	**<0.0001**
	OP50	0.3139	0.5753

[1] The time points and bacteria were analyzed separately.
[2] Bacteria, for which the difference between N2 and CB4856 was assessed with the Kruskall Wallis test, including the nematocidal *P. aeruginosa* PA14, and the control *E. coli* OP50. DF = 1 for all tests. Significant probabilities are given in bold. Significance level was adjusted using Bonferroni correction for multiple pairwise comparisons.
