# Supplementary material for: Contrasting invertebrate immune defense behaviors caused by a single gene, the Caenorhabditis elegans neuropeptide receptor gene npr-1
Source: BMC Genomics. 2016 Apr 11;17:280. doi: 10.1186/s12864-016-2603-8 (PMC4827197; doi:10.1186/s12864-016-2603-8)
Supplement: Additional file 12: — Table on the statistical results for the comparison of the N2 and CB4856 leaving behavior with that of the mutant strains towards P. aeruginosa and E. coli. (PDF 90 kb) [file 12864_2016_2603_MOESM12_ESM.pdf]

Additional File 11. Table on the statistical results for the comparison of the N2 and CB4856 leaving behavior with that of the mutant strains towards *P. aeruginosa* and *E. coli*

		**14 h**[1]		**24 h**[1]		**48 h**[1]	
**Comparison**[1]	**Bacteria**	***χ2***	***p***	***χ2***	***p***	***χ2***	***p***
N2 vs. *npr-1(ur89)*	PA14	10.664	**0.0011**	14.972	**0.0001**	14.961	**0.0001**
	OP50	1.466	0.226	0.285	0.5933	0.006	0.9367
N2 vs. *npr-1(ad609)*	PA14	12.695	**0.0004**	15.212	**<0.0001**	14.961	**0.0001**
	OP50	7.410	**0.0065**	4.962	0.0259	0.777	0.3781
N2 vs. *tyra-3(ok325)*	PA14	1.328	0.2490	0.359	0.5488	6.899	**0.0086**
	OP50	1.064	0.3022	2.924	0.0873	0.147	0.7009
N2 vs. CB4856	PA14	8.389	**0.0038**	15.212	**<0.0001**	13.096	**0.0003**
	OP50	2.031	0.1541	2.716	0.0993	1.587	0.2078
CB4856 vs. *npr-1(ur89)*	PA14	1.5598	0.2117	0.7917	0.3736	0.8517	0.3561
	OP50	0.2042	0.6514	2.8469	0.0915	1.1179	0.2904
CB4856 vs. *npr-1(ad609)*	PA14	3.8747	0.049	0	1	11.4147	**0.0007**
	OP50	2.5695	0.1089	0.5891	0.4427	0.0967	0.7559
CB4856 vs. *tyra-3(ok325)*	PA14	14.145	**0.0002**	15.397	**<0.0001**	7.949	**0.0048**
	OP50	0.175	0.6757	0.072	0.787	1.591	0.2071

[1] The analysis was performed separately for the pairwise comparisons, bacteria, and the three time points. The difference between *C. elegans* strains was assessed with the Kruskall Wallis test. The bacteria included the nematocidal *P. aeruginosa* PA14 and the control *E. coli* OP50. DF = 1 for all tests. Significant probabilities are given in bold. Significance level was adjusted using Bonferroni correction for multiple pairwise comparisons.
