# Supplementary material for: Contrasting invertebrate immune defense behaviors caused by a single gene, the Caenorhabditis elegans neuropeptide receptor gene npr-1
Source: BMC Genomics. 2016 Apr 11;17:280. doi: 10.1186/s12864-016-2603-8 (PMC4827197; doi:10.1186/s12864-016-2603-8)
Supplement: Additional file 13: — Table on the statistical results for the comparison of C. elegans survival rate on P. aeruginosa PA14 versus the control E. coli OP50. (PDF 74 kb) [file 12864_2016_2603_MOESM13_ESM.pdf]

Additional File 12. Table on the statistical results for the comparison of *C. elegans* survival rate on *P. aeruginosa* PA14 versus the control *E. coli* OP50

***C. elegans* strain**[1]	***χ2***	***p***
N2	114.72	**<0.0001**
CB4856	134.95	**<0.0001**
npr-1(ur89)	103.77	**<0.0001**
npr-1(ad609)	158.09	**<0.0001**

[1] We used the Kaplan-Meier method to calculate survival fractions and compared survival curves of each *C. elegans* strain on pathogenic *P. aeruginosa* PA14 versus the control *E. coli* OP50 with the help of a Log-rank test.
