# Supplementary material for: Contrasting invertebrate immune defense behaviors caused by a single gene, the Caenorhabditis elegans neuropeptide receptor gene npr-1
Source: BMC Genomics. 2016 Apr 11;17:280. doi: 10.1186/s12864-016-2603-8 (PMC4827197; doi:10.1186/s12864-016-2603-8)
Supplement: Additional file 14: — Table on the statistical results for the pairwise comparisons of C. elegans survival rates on P. aeruginosa PA14. (PDF 72 kb) [file 12864_2016_2603_MOESM14_ESM.pdf]

Additional File 13. Table on the statistical results for the pairwise comparisons of *C. elegans* survival rates on *P. aeruginosa* PA14

Comparison	$\chi 2$	*p*
N2 vs. CB4856	58.32	**<0.0001**
N2 *vs. npr-1(ur89)*	93.38	**<0.0001**
N2 *vs. npr-1(ad609)*	93.01	**<0.0001**

Survival curves were calculated with the Kaplan-Meier method and the Log-rank test was used for each pairwise comparison separately. Significant probabilities are given in bold. Significance level was adjusted using Bonferroni correction for multiple pairwise comparisons.
